# Modulation of oncogenic miRNA biogenesis using functionalized polyamines

**DOI:** 10.1038/s41598-018-20053-5

**Published:** 2018-01-26

**Authors:** Cathy Staedel, Thi Phuong Anh Tran, Julie Giraud, Fabien Darfeuille, Audrey Di Giorgio, Nicolas J. Tourasse, Franck Salin, Philippe Uriac, Maria Duca

**Affiliations:** 1 Université de Bordeaux, INSERM, ARNA, 33076 Bordeaux, France; 2 Université Côte d’Azur, CNRS, ICN, 06107 Nice, France; 3 Université de Bordeaux, INSERM, BARITON, 33076 Bordeaux, France; 4 Université de Bordeaux, INRA, BIOGECO, 33612 Cestas, France; 5 Université de Rennes 1, CNRS, ISCR, 35042 Rennes, France

## Abstract

MicroRNAs are key factors in the regulation of gene expression and their deregulation has been directly linked to various pathologies such as cancer. The use of small molecules to tackle the overexpression of oncogenic miRNAs has proved its efficacy and holds the promise for therapeutic applications. Here we describe the screening of a 640-compound library and the identification of polyamine derivatives interfering with *in vitro* Dicer-mediated processing of the oncogenic miR-372 precursor (pre-miR-372). The most active inhibitor is a spermine-amidine conjugate that binds to the pre-miR-372 with a K_D_ of 0.15 µM, and inhibits its *in vitro* processing with a IC_50_ of 1.06 µM. The inhibition of miR-372 biogenesis was confirmed in gastric cancer cells overexpressing miR-372 and a specific inhibition of proliferation through de-repression of the tumor suppressor LATS2 protein, a miR-372 target, was observed. This compound modifies the expression of a small set of miRNAs and its selective biological activity has been confirmed in patient-derived *ex vivo* cultures of gastric carcinoma. Polyamine derivatives are promising starting materials for future studies about the inhibition of oncogenic miRNAs and, to the best of our knowledge, this is the first report about the application of functionalized polyamines as miRNAs interfering agents.

## Introduction

MicroRNAs (miRNAs) are single-stranded non-coding RNAs bearing an essential role in the regulation of gene expression^1^. Upon binding to the 3′ untranslated regions (3′-UTR) of specific target messenger RNAs (mRNAs), these short RNAs (22–24 nucleotides) inhibit mRNA translation and induce mRNA degradation thus leading to inhibition of protein expression^2^. A single miRNA has hundreds of target genes and this class of small RNAs represents a powerful way of gene regulation. The miRNA biogenesis process starts with the transcription of a long primary RNA of variable size (pri-miRNA), which is processed in the nucleus by the enzyme Drosha into a shorter (ca. 70 nucleotides) stem-loop-structured precursor (pre-miRNA)^3^. This latter is exported to the cytoplasm, where it becomes a substrate for the Dicer enzyme. Processing by Dicer produces the mature miRNA, which is loaded onto the RNA-induced silencing complex (RISC) and subsequently targets complementary sequences on mRNAs^4^. While this physiological process is absolutely essential for cellular homeostasis, it also represents an extremely delicate equilibrium, whose deregulation, i.e. underexpression or overexpression of some miRNAs, has been directly linked to a wide number of pathologies such as cancers^5^. Over the last few years, relevant strategies have been developed in order to inhibit overexpressed miRNAs (oncogenic miRNAs) or induce the biogenesis of underexpressed miRNAs (tumor suppressor miRNAs) in tumor cells^6^. The most intuitive and specific approach is based on the use of oligonucleotides that can either replace the lacking tumor suppressor miRNA or specifically pair with the oncogenic miRNA thus preventing its interaction with the targeted mRNAs^7^. However, the oligonucleotide-based strategies still suffer from a number of limitations for their therapeutic applications mainly due to poor pharmacodynamic and pharmacokinetic properties of oligonucleotides. Another approach is based on the use of small molecules able to interfere with one of the steps of miRNA biogenesis^8,9^. Most of the reported miRNA-interfering small-molecule agents were identified after the screening of large libraries and led to the discovery of specific inhibitors of oncogenic miRNA production. As an example, diazobenzene derivatives were selected as specific inhibitors of miR-21 transcription after the screening of a chemical library^10^. Furthermore, guanidylated neomycin B and kanamycin A were discovered after two-dimensional combinatorial screening (2-DCS) as specific miR-10b inhibitors upon binding to pri-miRNA and inhibition of Drosha-mediated cleavage^11^. Some of the currently identified small-molecule inhibitors of miRNAs have also been discovered upon more rational approaches. As an example, Disney and co-workers applied a sophisticated methodology called Inforna allowing for the discovery of highly specific and efficient ligands of miRNA precursors that are able to inhibit miRNA biogenesis inside cells^12^. These studies were crucial for increasing the general knowledge about the possibility to interfere with miRNAs using small compounds and for demonstrating that it is actually possible to specifically block the production of a particular miRNA with this approach.

Concomitantly with the identification of compounds bearing high selectivity for a particular miRNA, a number of compounds targeting a range of different miRNAs in a non-specific manner have also been identified. Contrary to what could be expected, some of these non-selective compounds also showed interesting and specific biological activities^13–15^. A noteworthy example is AC1MMYR2, a non-specific inhibitor of miR-21 biogenesis discovered after *in silico* high throughput screening^15^. This compound affects eleven additional miRNAs, but despite this lack of selectivity, the dose-dependent effect seemed to occur only on miR-21 upon inhibition of its processing by Dicer^16,17^.

During our ongoing efforts toward the discovery of new RNA ligands able to interfere with miRNA biogenesis, we also designed new multimodal compounds showing a specific biological activity^18,19^. These RNA ligands are inhibitors of miR-372 and miR-373 production, two miRNAs that are oncogenic in several types of cancers^20^. These compounds were synthesized upon conjugation of artificial nucleobases to aminoglycosides leading to compounds that bind with high affinity to stem-loop structured pre-miR-372 and pre-miR-373. This led to a decrease in miR-372 and miR-373 levels and to the restoration of normal translation of their common target, the tumor suppressor LATS2 protein, a serine-threonine kinase involved in cell cycle regulation^21^. Despite the fact that these compounds lack selectivity and are able to inhibit the biogenesis of various miRNAs, some of them bear an antiproliferative effect, which is specific for gastric cancer cells overexpressing the targeted miRNAs.

In an effort to identify original and unexpected RNA binding structures able to interfere with oncogenic miRNA biogenesis, we describe here the screening of a 640-member library from the French National Chemical Library. Initial cell-free assays allowed us to select three compounds that inhibit Dicer processing *in vitro* upon binding to targeted pre-miR-372: these three molecules were all polyamine derivatives. One of them showed a promising antiproliferative activity in gastric cancer cells overexpressing targeted miR-372 and was further studied for its intracellular molecular mechanism of action. The obtained results showed that functionalized polyamines are extremely promising RNA binders able to interfere with miRNA biogenesis upon binding to miRNA precursors and to induce a specific inhibition of proliferation in cancer cells overexpressing targeted miRNA.

## Results and Discussion

### Cell-free screening of a 640-member library for inhibition of Dicer-mediated miR-372 processing

The aim of this work was to identify new scaffolds for RNA binding and inhibition of miRNA biogenesis upon screening of a medium-sized, diversified chemical library. The French National Chemical Library (chimiotheque-nationale.cn.cnrs.fr) is a collection of more than 60,000 synthetic molecules, natural compounds and natural extracts available in French academic laboratories for a targeted or a systematic biological evaluation. In this work, we decided to start with the screening of a smaller set of 640 compounds representative for the chemical diversity of the entire collection. The approach consisting in targeting biologically relevant RNAs, such as oncogenic miRNAs, using small molecules is based on the fact that most of these targets are organized in a stem-loop structure associating single-stranded regions and double-stranded regions, and offering in its three-dimensional conformation the possibility for ligand binding^8,9,22^. In miRNAs biogenesis, both pri-miRNA and pre-miRNA could thus represent potential targets for this approach. Since the pre-miRNA sequence is also included in the pri-miRNA one, we and others took advantage of the shortest sequence of pre-miRNAs for the development of cell free assays in the aim of screening and identifying compounds that could eventually bind one or both precursors and inhibit the entire biogenesis pathway in a selective manner^23,24^. The initial screening was performed using a previously reported and validated cell-free assay^18,19^. In this assay, the targeted pre-miRNA was double labeled with a fluorophore (fluorescein) and a quencher (dabcyl) at the 3′ and 5′ end, respectively. In the presence of human recombinant Dicer ribonuclease, this latter cleaves the pre-miRNA at defined sites and fluorescence appears. If a RNA ligand efficiently binds to the structured pre-miRNA and inhibits the cleavage by Dicer, no fluorescence is detected. Initially, we decided to focus our attention on the inhibition of miR-372 that is overexpressed in some cancers, while it is undetectable in healthy cells^20,21^. The compounds were thus tested in a blind screening at a concentration of 0.1 mg/mL for pre-miR-372 processing inhibition. The screening led to the identification of eight compounds able to inhibit by more than 50% Dicer-mediated cleavage.

The eight active compounds were then studied for their binding affinity for pre-miR-372 using a fluorescence-based assay allowing for the evaluation of the dissociation constant (K_D_). Only three of them (compounds **PA-1**, **PA-2** and **PA-3**, Table 1 and Supplementary Fig. S1A) were able to bind the RNA target, the other five likely being non-specific Dicer inhibitors. Compound **PA-1** is the best ligand showing K_D_ value of 0.15 µM, while **PA-2** shows slightly higher K_D_ value. Compound **PA-3** is the weakest binder. These data were combined with IC_50_ measurements performed using the same cell-free assay employed for the initial screening, over a range of inhibitor concentrations (Table 1 and Supplementary Fig. S1B). According to its good affinity for the RNA target, **PA-1** also shows the best IC_50_ value (1.06 µM) while compounds **PA-2** and **PA-3** show a 10 times lower activity. **PA-2** and **PA-3** bear similar K_D_ values but **PA-2** is twice more efficient in inhibiting pre-miR-372 processing. This lack of correlation between K_D_ and IC_50_ is probably due to differences in each ligand binding site that is known to be determining for efficient inhibition as observed previously for other series of inhibitors^19,25^.

**Table 1:** IDs and chemical structures of compounds identified after the screening of Dicer inhibition: dissociation constants (K_D_) and inhibition activity (IC_50_) measured in the presence of pre-miR-372.

ID	Structure	pre-miR-372	
		K_D_ (µM)^a^	IC_50_ (µM)^b^
**PA-1**		0.15 ± 0.01	1.06 ± 0.09
**PA-2**		0.53 ± 0.07	16.3 ± 0.2
**PA-3**		1.15 ± 0.3	12.7 ± 0.1

Related to Tables S1 and S2.

^a^Binding affinities (dissociation constants, K_D_) were evaluated using 5′-FAM-pre-miR-372 beacon in Buffer A and represent the average of three independent experiments with their standard deviation. ^b^IC_50_ experiments were performed in the presence of 50 nM of 3′-dabcyl-5′-FAM-pre-miR-372 or 3′-dabcyl-5′-FAM-pre-miR-373 beacon and 0.25U of human recombinant Dicer in Buffer A and represent the average of three independent experiments with their standard deviation.

### Functionalized polyamines as inhibitors of miRNA biogenesis

The three hits displaying both affinity for the targeted pre-miRNA and inhibition activity for their Dicer-mediated processing turned out to be conjugates of natural polyamines (Table 1): **PA-1** derives from the conjugation of spermine with a heterocyclic amidine^26^, **PA-2** (*N*^1^,*N*^4^-bis(4-aminobutyl)-1,4-butanediamine) represents a homologation of spermine^27^ and **PA-3** is a conjugate between putrescine and (+)-usnic acid (a dibenzofuran moiety)^28^. Polyamines (putrescine, spermidine, spermine and related structures) are natural compounds ubiquitously distributed in eukaryotic cells and essential for growth and differentiation. A wealth of literature is devoted to these endogenous compounds that bear a large number of intracellular roles under physiological conditions. For example, polyamines are involved in maintaining the native structure of several biological macromolecules while affecting the activity of others through tightly regulated concentration-dependent processes^29^. Polyamines can also be involved in pathological processes. Indeed, cancer cells frequently require high polyamine levels due to their enhanced growth. For this reason, various therapeutic strategies have been envisaged in order to inhibit production, uptake or functions of polyamines. One possible approach is the synthesis of polyamine analogs that resemble the native ones sufficiently to repress polyamine production by negative feedback or uptake by inhibition of the polyamine transport system (PTS), but deprived of the ability to support cell growth^30^. It is also important to note that numerous works have suggested that polyamines function as modulators of RNA structure upon binding and induction of a conformational change that stabilizes the target. The interactions can be formed non-specifically with the phosphates of the RNA backbone, but they can also engage specific binding with distinct nucleic acid residues in a defined binding pocket formed in the secondary structure of RNA macromolecules^31^. The identification of these compounds as promising ligands of miRNA precursors and as inhibitors of miRNA biogenesis was thus fully significant and stressed an additional and yet unexplored interaction of polyamine conjugates with these biologically relevant RNAs.

### Effect of PA-1, PA-2 and PA-3 on gastric adenocarcinoma cells

**Figure 1:**
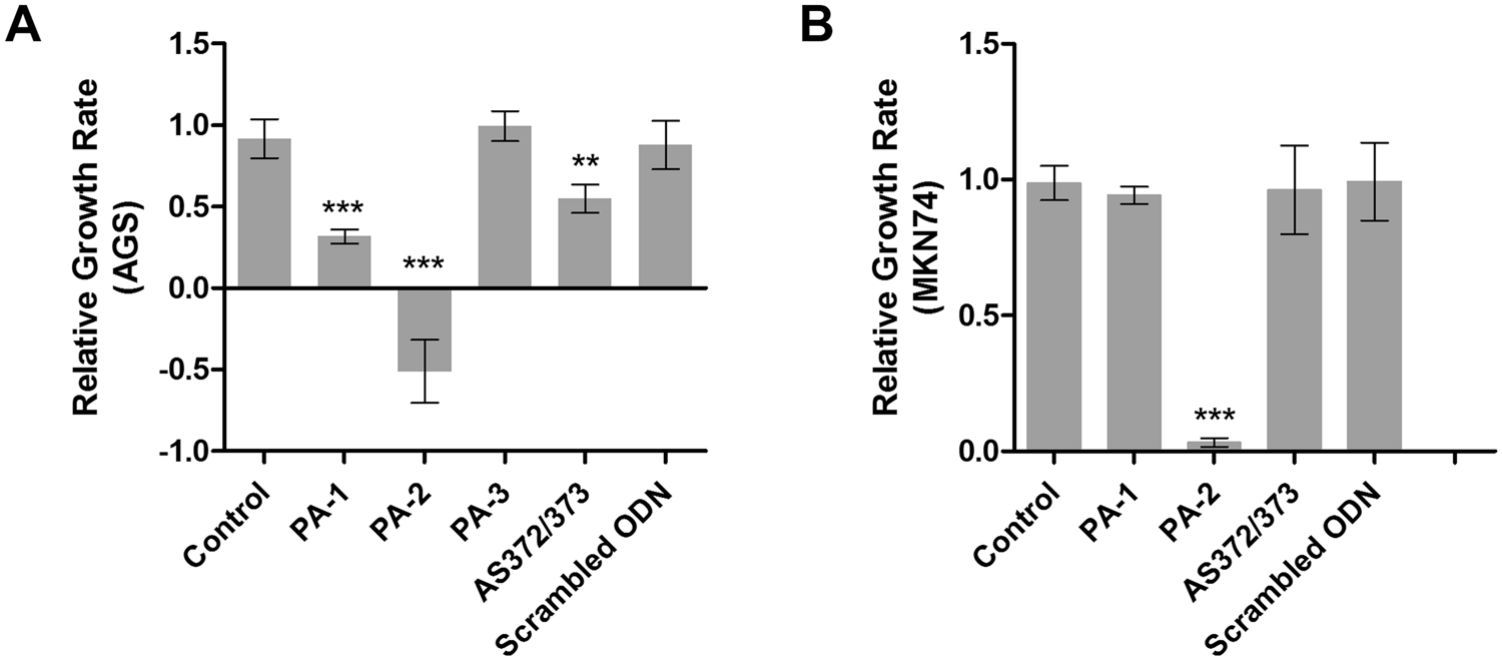
Relative growth rate of AGS cells (**A**) expressing high miR-372 and miR-373 levels and MKN74 cells (**B**) in the presence of compounds **PA-1**, **PA-2** and **PA-3** (50 µM) or upon transfection with antimiR-372-373 oligonucleotide or scrambled oligonucleotide (10 nM). Growth was determined by (A_492nm_ at day 4− A_492nm_ at day 2)/A_492nm_ at day 2. Bars represent the mean ± standard deviation (SD) of growth rate data compared to those of untreated cells. ***p < 0.001, **p < 0.01.

Before going further in the study of the three selected compounds, we assessed their biological activity on the human gastric carcinoma cell line AGS, which expresses high levels of both miR-372 and -373 and depends on them for its growth^32^. Each compound was added in the culture medium at a concentration of 50 μM and cell viability was measured after 4 days (Fig. 1A). This viability assay was also performed on the MKN74 gastric carcinoma cell line that expresses 100 times less miR-372 (Fig. 1B). Whereas **PA-1** inhibits AGS cells growth by 70%, a rate higher than the one achieved by anti-miR-372/373 antisense oligonucleotides (AS372/373), it has no effect on MKN74 cells at the tested concentration. **PA-2** is highly toxic for both cell lines suggesting that the toxicity of this compound may be related to strong off-target effects. Finally, **PA-3** does not affect the viability of either AGS or MKN74 cells. Altogether, these results show that **PA-1** induces a selective inhibition of proliferation for cells overexpressing miR-372 since no effect could be observed in cells that express low levels of this miRNA as confirmed not only by comparing AGS to MKN74 cells but also to three different cell lines (MKN7, MKN28 and NCI-N87) that do not express miR-372 (Supplementary Fig. S2). It is important to note that **PA-1** appears less toxic than the natural unconjugated spermine, which rapidly killed both AGS and MKN74 cells (Supplementary Fig. S3), suggesting that polyamine derivatives bearing cyclic and heteroaromatic substitutions may endow interesting biological activities. The low K_D_ and IC_50_ combined to the selective cancer cell growth inhibitory activity prompted us to choose **PA-1** for further investigations.

### PA-1 inhibits miR-372 biogenesis and cell growth of human gastric carcinoma cells in culture

**Figure 2:**
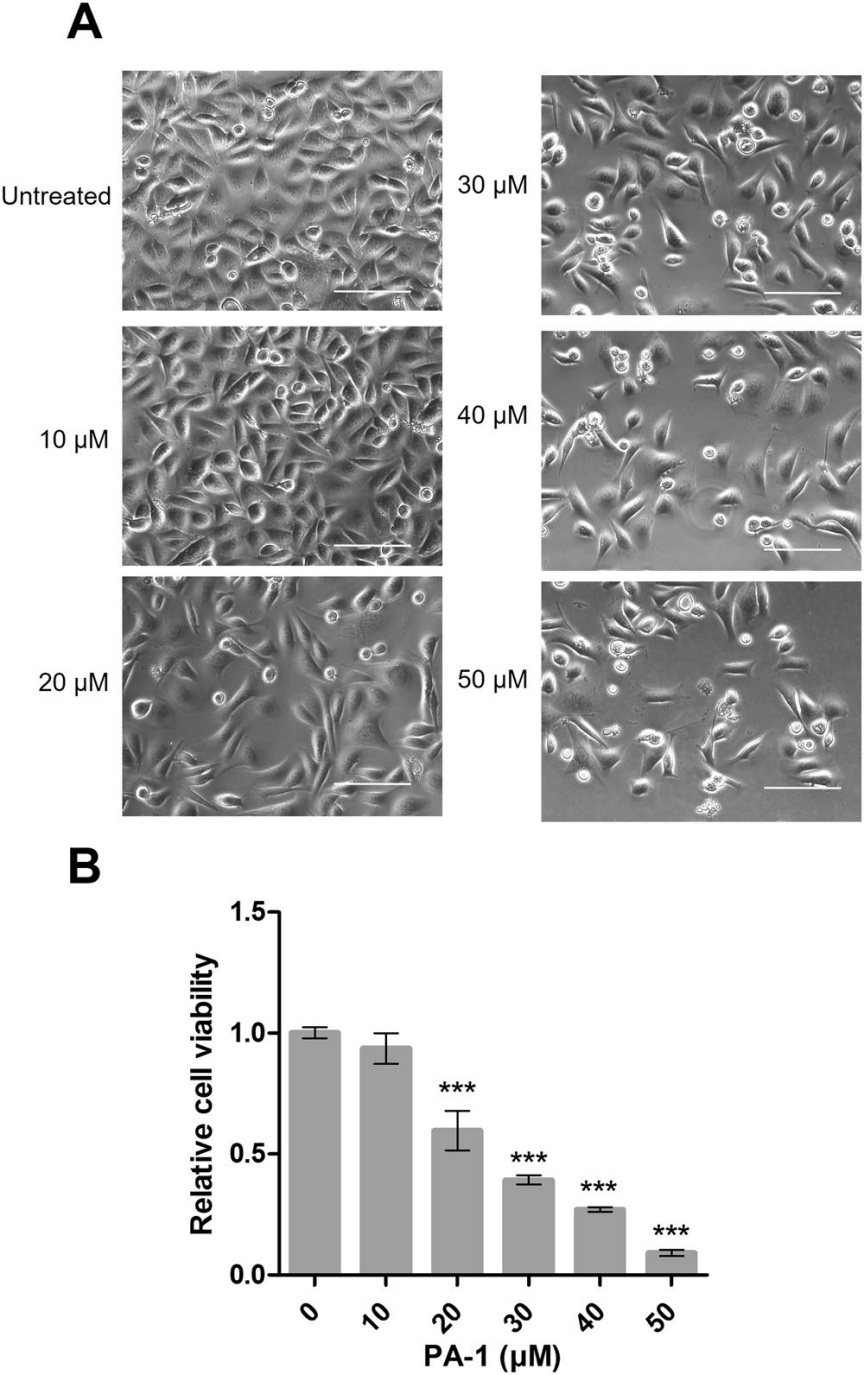
(**A**) Phase contrast microscopic observation of living AGS cells without treatment and after treatment with increasing concentrations of compound **PA-1** for 4 days using an inverted-light microscope (Zeiss) equipped with a 20× objective. Bars = 100 µm. (**B**) Relative growth rate of AGS cells in the presence of growing concentrations of **PA-1**.

**Figure 3:**
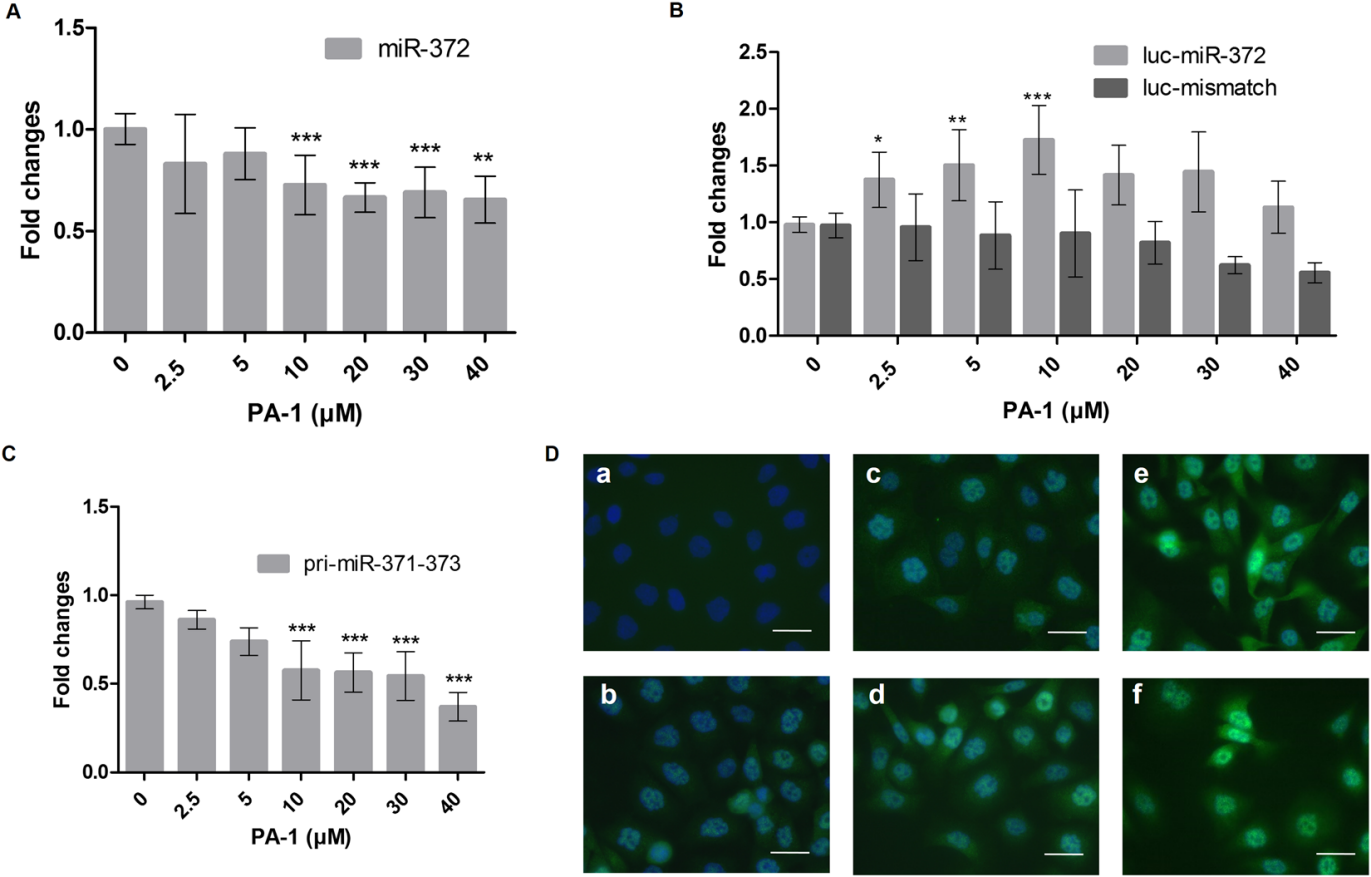
(**A**) RT-qPCR quantification of miR-372 after a 4-day treatment of AGS cells in the presence of increasing doses of **PA-1**. Bars represent the mean ± SD of miRNA expression normalized to both RNU49 and snU6 and compared to untreated cells (n = 4). (**B**) Relative luciferase activity of the miR-372 reporter pGL4-PM372 (luc-miR-372) or control reporter (luc-mismatch) transfected into AGS cells previously treated for 3 days with compound **PA-1** at increasing concentrations. Bars represent the mean ± SD of firefly luciferase activities of each reporter compared to that of untreated cells (n = 4). (**C**) RT-PCR quantification of pri-miR-371-372-373 after a 4-day treatment by **PA-1**. Bars represent the mean ± SD of pri-miR-371-373 expression normalized to the housekeeping genes and compared to untreated cells (n = 6). (**D**) Subcellular localization of LATS2 protein in AGS cells using immunofluorescence microscopy. a) Negative control, b) untreated cells, c) 24 h treatment with **PA-1** at 10 µM, d) 72 h treatment with **PA-1** at 10 µM, e) 24 h treatment with **PA-1** at 25 µM and f) 72 h treatment with **PA-1** at 25 µM. LATS2 appears in green fluorescence and nuclei are revealed by Hoechst staining (blue); LATS2 was labeled using a rabbit anti-human LATS2 antibody followed by an Alexa 488-labeled anti-rabbit antibody and appears in green. Bars = 50 µm.

To assess whether **PA-1** could represent an efficient compound to interfere with miRNA biogenesis in cancer cells, we performed deeper studies about its intracellular effects. First, we observed that **PA-1** inhibits AGS cell growth in a dose-dependent manner (Fig. 2A). The quantification of cell viability demonstrated that the inhibition of proliferation starts at a concentration over 10 μM and that **PA-1** bears a IC_50_ in the range of 20 µM (Fig. 2B). As no cell debris could be observed in the culture medium, we assumed that the effect of **PA-1** was rather cytostatic than cytotoxic. Indeed, cell cycle analysis by flow cytometry showed an increased proportion of G0/G1 phase cells at the detriment of S phase cells upon 48 h treatment with 25 µM **PA-1**, compared to untreated or 10 µM **PA-1**-treated cells (Supplementary Fig. S4A). No sub-G1 (apoptotic) cell population was visible. Moreover, immunostaining of the cyclin-dependent kinase inhibitor CDKN1A/p21^cip^ showed an increased number of p21-positive, unfragmented nuclei upon **PA-1** treatment compared to control cells (Supplementary Fig. S4B). Furthermore, p21 mRNA was increased in a **PA-1** dose-dependent manner, while the cell growth marker PCNA (expressed during S phase) decreased and the programmed cell death marker (PDCD4) remained unchanged (Supplementary Fig. S4C). Finally, the growth inhibitory effect of **PA-1** was reversible, since removal of **PA-1** treatment allowed recovery of cell growth ability (Supplementary Fig. S4D). All combined, these data indicate that **PA-1** inhibited the growth of AGS cell by arresting them at the G1/S transition of the cell cycle. In previous studies, only a modest activity for this compound was reported in cancer cells (L1210) since IC_50_ was in the high micromolar range^26^. Noteworthy, **PA-1** inhibits the proliferation of AGS cells in a greater amount compared to antimiR oligonucleotides directed against miR-372 and miR-373 that were able to induce at most 50% inhibition of cell growth under the same conditions (Fig. 1A and^32^). In agreement with our *in vitro* studies demonstrating inhibition of pre-miR-372 processing by **PA-1**, this compound decreased mature miR-372 levels in AGS cells by 35% depending on the dose (Fig. 3A). This result obtained by RT-PCR quantification of miR-372 was validated using a miR-372-luciferase reporter (luc-miR-372) harboring a miR-372 binding site downstream to the firefly *luciferase* coding sequence: upon binding to the luciferase mRNA transcribed from this construct, miR-372 post-transcriptionally represses luciferase expression under basal conditions. Hence, the decrease in miR-372 levels induced by **PA-1** is accompanied by a dose-dependent de-repression of the luciferase reporter in the limits of 2.5–20 μM concentrations (Fig. 3B). In this range of concentrations of **PA-1**, the increased activity of the luciferase reporter is specific to the miR-372 decreased levels, since a control reporter (luc-mismatch) carrying a mismatched miR-372 binding site was not affected. Nevertheless, this control reporter exhibited a significant inhibition at doses over 30 μM, suggesting that **PA-1** has off-target effects at higher concentrations. MiR-372 belongs to a unique cluster in the human genome along with miR-371 and miR-373 and the decrease of endogenous mature miR-372 level may also involve an interaction of **PA-1** with the pri-miR-371–372–373. Indeed, a decrease in pri-miR-371-372-373 relative to housekeeping gene mRNAs is observed in response to increasing doses of **PA-1** (Fig. 3C), likely contributing to miR-372 drop. This effect is likely due to pri-miRNA instability upon **PA-1** binding and is in agreement with an interaction of **PA-1** with a functional site of pri-miR-371-372-373 and/or pre-miR-372 that is responsible for the inhibition of miRNA production since the pre-miRNA sequence and structure is also included in the pri-miRNA one.

Given that **PA-1** decreases levels of mature miR-372, we studied the downstream cellular response to **PA-1** and in particular one of the main targets of this oncogenic miRNA: the tumor suppressor LATS2 protein. LATS2 immuno-fluorescent labeling depicted in Fig. 3D shows that under basal conditions, AGS weakly expressed LATS2, which is repressed by high miR-372/373 levels. Upon treatment with **PA-1**, LATS2 accumulates in an increasing number of cells, mainly in the nucleus, in dose and time-dependent manners, along with decreased cells number. Importantly, the accumulation of nuclear LATS2 protein in **PA-1**-treated cells is not due to enhanced *lats2* transcription, since LATS2 mRNA levels were the same in treated and untreated cells as measured by RT-qPCR (0.856 ± 0.093 at 10 µM and 0.869 ± 0.152 at 25 µM). Noteworthy, the levels of expression of LATS2 in **PA-1**-treated cells are similar to those obtained using the antimiR oligonucleotides directed against miR-372 and miR-373^32^. Thus, even if we cannot exclude that **PA-1** also affects the PTS in AGS cells, especially at the highest concentrations, our data suggest that **PA-1** reaches its target in a cell environment and interferes with miR-372 processing and hence with its oncogenic function.

### PA-1 affects additional miRNAs in the miRnome of AGS cells

**Figure 4:**
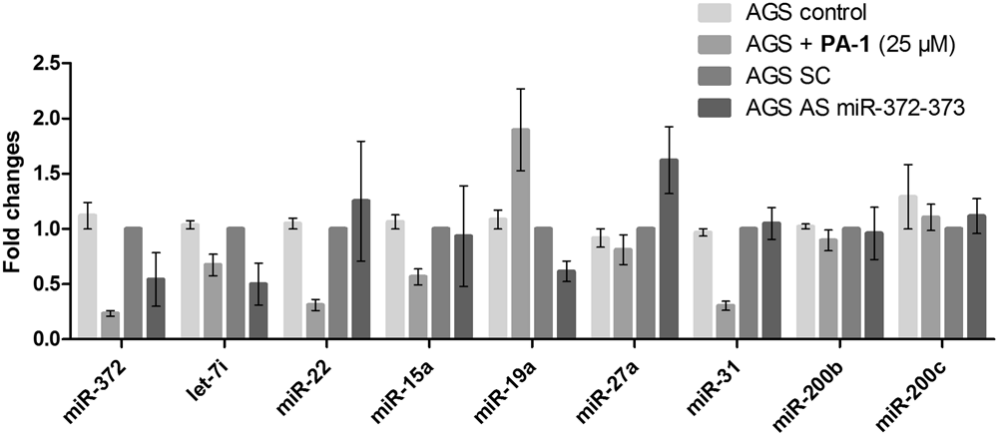
RT-qPCR quantification of miRNA after a 4-day treatment with 25 μM of compound **PA-1** or transfection with 10 nM antimiR-372-373 oligonucleotide (AS372/373) or scrambled oligonucleotide (SC). Bars represent the mean ± SD of miRNA expression normalized to both RNU49 and snU6 and compared to untreated or SC cells.

To assess whether **PA-1** could affect additional miRNAs, which could contribute to the growth inhibition effect, we performed a complete miRNA profiling of the AGS cells treated twice with 25 μM of **PA-1** and compared it to that of untreated cells. A cDNA library was generated from cell RNAs < 200 nucleotides for each sample and submitted to NGS technology^33,34^. A total of 400,000 reads were analyzed, among which mature miRNAs were identified (Supplementary Table S1). AGS express about 70 detectable mature miRNAs including the abundant miR-372 and miR-21 as previously reported^32^. Whereas the miR-372 number of reads was found to be decreased by 50% in **PA-1**-treated cells as compared to untreated cells, other miRNAs were affected: the miR-22, miR-19a, let-7i, miR-15a, miR-31 and miR-192 number of reads were decreased more than 50% while miR-371-3p and miR-373 by 40% and 20%, respectively (Supplementary Fig. S5A). In addition, some miRNAs were found to be upregulated, such as miR-93, miR-30b, miR-30d or mir-106b, since the number of reads was increased more than twice in **PA-1**-treated cells compared to untreated cells (Supplementary Fig. S5B). The changes in expression of some of these miRNAs were confirmed by RTqPCR on the same samples used for the miRnome analysis (Supplementary Fig. S6). We then wondered whether the repression of theses miRNAs was a direct consequence of **PA-1** interaction with their precursors (pre-miRNAs or pri-miRNAs) or rather an indirect effect following miR-372 repression. Therefore, we compared the expression of some of the affected miRNAs in AGS cells either treated with 25 μM of **PA-1** or transfected with 10 nM antisense anti-miR-372/373 (AS372/373) for 4 days (Fig. 4). Both **PA-1** and AS372/373 decreased the expression of miR-372 relative to untreated cells or scrambled oligo-transfected cells, respectively. This further confirms the important role of the inhibition of miR-372 in the biological effect of **PA-1**. The decrease in miR-372 levels is in agreement with the one observed in previous quantification studies (Fig. 3A) and was even better thanks to two following treatments of AGS cells with compound **PA-1**. The decrease of miR-22, miR-15a and miR-31 levels was also confirmed in the presence of **PA-1** while AS372/373 had no effect. This suggests that **PA-1** can also directly affect the biogenesis of these miRNAs. Finally, while let-7i is decreased with both **PA-1** and AS372/373, the other evaluated miRNAs were not affected by **PA-1**. In conclusion, miRnome analysis showed that **PA-1** does not affect the general miRNA biosynthesis machinery, but decreases or enhances selectively some miRNAs. Among the miRNAs downregulated by **PA-1**, it has to be noted that miR-31 also targets LATS2^35^. This latter is also one of the predicted targets of miR-15a and miR-22 (as for mirdb.org and targetscan.org)^36,37^. Therefore, some of the affected miRNAs likely participates to the cell growth inhibition of **PA-1**-treated AGS cells. However, miR-372 clearly bears a pivotal role in this inhibition effect, since the **PA-1**-insensitive MKN74, MKN7, MKN28 cell lines express miR-15a and miR-22, while they do not express miR-372 and faintly miR-31, and the slightly sensitive NCI-N87 cell line expresses miR-31 (Supplementary Fig. S2B). The fact that these cells are not or weakly affected by **PA-1** treatment suggests that the inhibition of proliferation observed in AGS cells is directly linked to an effect on miR-372 production. This was further confirmed after lipotransfection of a mimic of mature, double stranded miR-372 (mim372) that was indeed able to prevent **PA-1**-mediated inhibition of AGS cells growth, while it did not affect cell growth of untreated cells, compared to a double stranded 21-mer RNA (siControl) (Supplementary Fig. S7).

### Antitumor activity of PA-1 in patient-derived gastric carcinoma cells grown *ex vivo* as tumorspheres

Tumors in patients are hierarchically organized from cancer stem cells (CSCs), at the origin of tumor initiation, heterogeneity, and propagation^38^. CSCs resist conventional therapy and are at the origin of tumor relapse and metastasis. The CSC reservoir has been characterized in gastric carcinoma (GCs)^39^, which is currently the third cancer-related cause of death worldwide. It mostly affects elderly and, being asymptomatic, is usually detected too late; it has therefore a bad prognosis. Moreover, there is no targeted therapy for GC so far.

**Figure 5:**
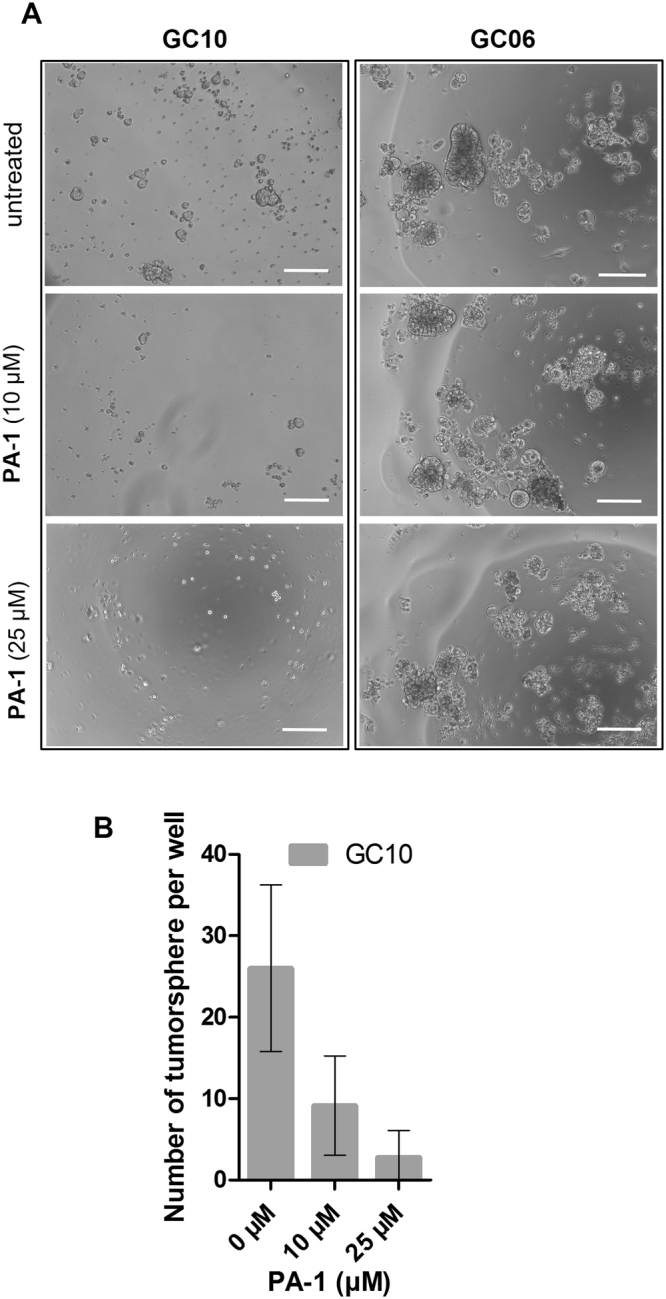
(**A**) Phase contrast microscopic observations of living GC tumorspheres without treatment or after in the presence of compound **PA-1** at 10 and 25 µM for 5 days, using an inverted-light microscope (Zeiss) equipped with a 10 × objective. Bars = 100 µm. (**B**) Numeration of GC10 tumorpheres per well in the presence of growing concentrations of **PA-1** measured at day 5 of treatment. Bars represent the mean ± SD of tumorphere number compared to untreated cells (out of 6 experiments, with 10 wells per condition).

As demonstrated so far, **PA-1** harbors a selective effect for targeted AGS cells, since it did not affect the growth of MKN74 cells that express very low miR-372 levels. This selective activity was further assessed on patient-derived GC cells that were dissociated from freshly collected patients-derived xenografts established in immunodeficient mice and submitted to the *in vitro* tumorsphere growth assay^40^ in the presence of increasing **PA-1** concentrations. Indeed, three-dimensional culture conditions in serum-free media allow the formation of a tumor cell spheroid (tumorsphere) from a single growing cell. Tumorspheres are considered to be closer to physiological conditions than monolayer cultures and reveal the presence within the tumor of CSCs^39^. While both GC10 and GC06 express miR-200b, which is a hallmark of the epithelial origin of these cells, miR-372 is homogenously expressed in GC10 but only in some cell clusters in GC06^39^. As illustrated in Fig. 5A, GC cells isolated from these primary tumors formed more or less compact tumorspheres. We evaluated the effects of **PA-1** on their ability to initiate tumorspheres: after 5 days of culture in the presence of **PA-1**, GC10 was markedly hampered to initiate tumorspheres, the number of which was found to be decreased in the presence of increasing doses of **PA-1** (Fig. 5A and B), whereas GC06 viability was unaffected. These results further confirm the selective activity of **PA-1** and also reveal its potential to target the CSC reservoir in GC and to prevent their characteristic self-renewal. Therefore, **PA-1**, which is able to target miR-372, is clearly a promising starting point for future anticancer strategies applicable to other cancer subsets depending on this oncomiR for their growth.

### Binding studies reveal a selective interaction of PA-1 with pre-miR-372 internal bulge and terminal loop

In order to gain better insights about the mechanism of action of **PA-1**, we first studied the selectivity of this compound for pre-miR-372 in the presence of a large excess (100 eq.) of tRNA or DNA. These latter are common nucleic acid competitors highly abundant inside cells, thus representing possible undesired targets for the studied compounds. The obtained values (K′_D_ and K′′_D_, for tRNA and DNA competition experiments, respectively, in Supplementary Table S2) showed that **PA-1** is selective for pre-miR-372 in the presence of both tRNA and DNA duplex (ratios K′_D_/K_D_ and K′′_D_/K_D_ between 1.0 and 1.2). Next, we evaluated IC_50_ values for the inhibition of pre-miR-372 processing by Dicer in the presence of HEK93 cell lysates instead of the recombinant enzyme (IC′_50_ in Table S3). Cell lysates indeed contain other potential competitors (both proteins and nucleic acids) that could interfere with the inhibition activity thus representing an appropriate control for the selectivity of the tested compounds. All values were higher than the ones measured in the presence of recombinant Dicer enzyme indicating a slight loss of activity. However, **PA-1** showed IC'_50_/IC_50_ ratio of 2.7 for the tested pre-miRNA thus confirming a promising selectivity as previously observed for tRNA and DNA competition. **PA-1** thus appears selective for pre-miR-372 both in competition binding experiments and in the presence of cell lysates instead of recombinant Dicer enzyme.

**Figure 6:**
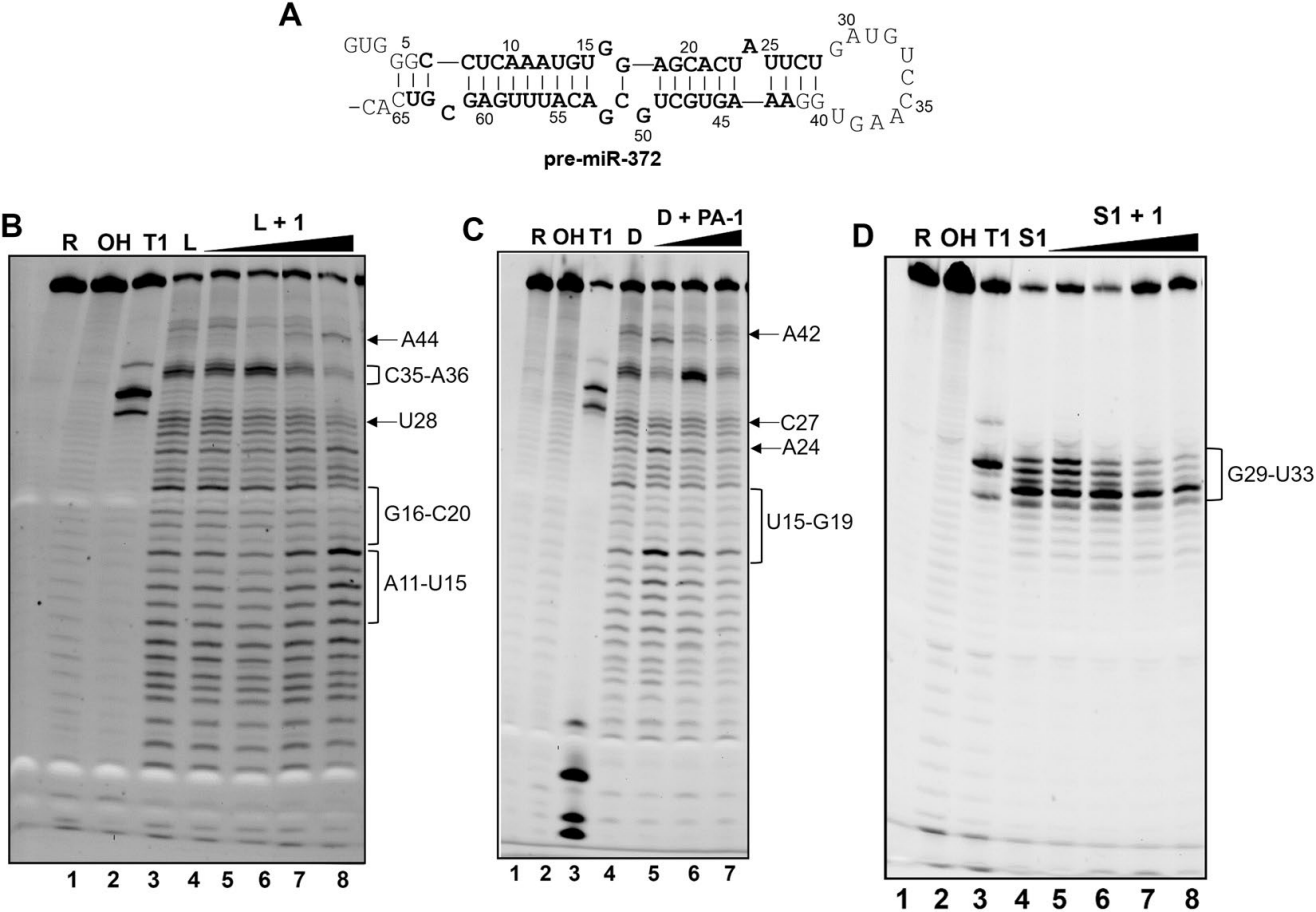
Footprinting analyses. (**A**) Sequence and secondary structure of pre-miR-372; (**B**) Lead(II) acetate probing of pre-miR-372 in the presence of 0, 1, 5, 10 and 50 µM of compound **PA-1** (lanes 4–8). (**B**) Human Recombinant Dicer probing of pre-miR-372 in the presence of 0, 0.5, 1 and 5 µM of compound **PA-1** (lanes 4–7). (**C**) RNase S1 probing of pre-miR-372 in the presence of 0, 1, 5, 10 and 50 µM of compound **PA-1** (lanes 4–8). In all gels, lane 1 represents intact RNA, lane 2 and lane 3 represent the alkaline hydrolysis ladder and T1 digestion ladder, respectively. Gels were cropped for clarity and complete gels are presented in Supporting Information Fig. S9.

**Figure 7:**
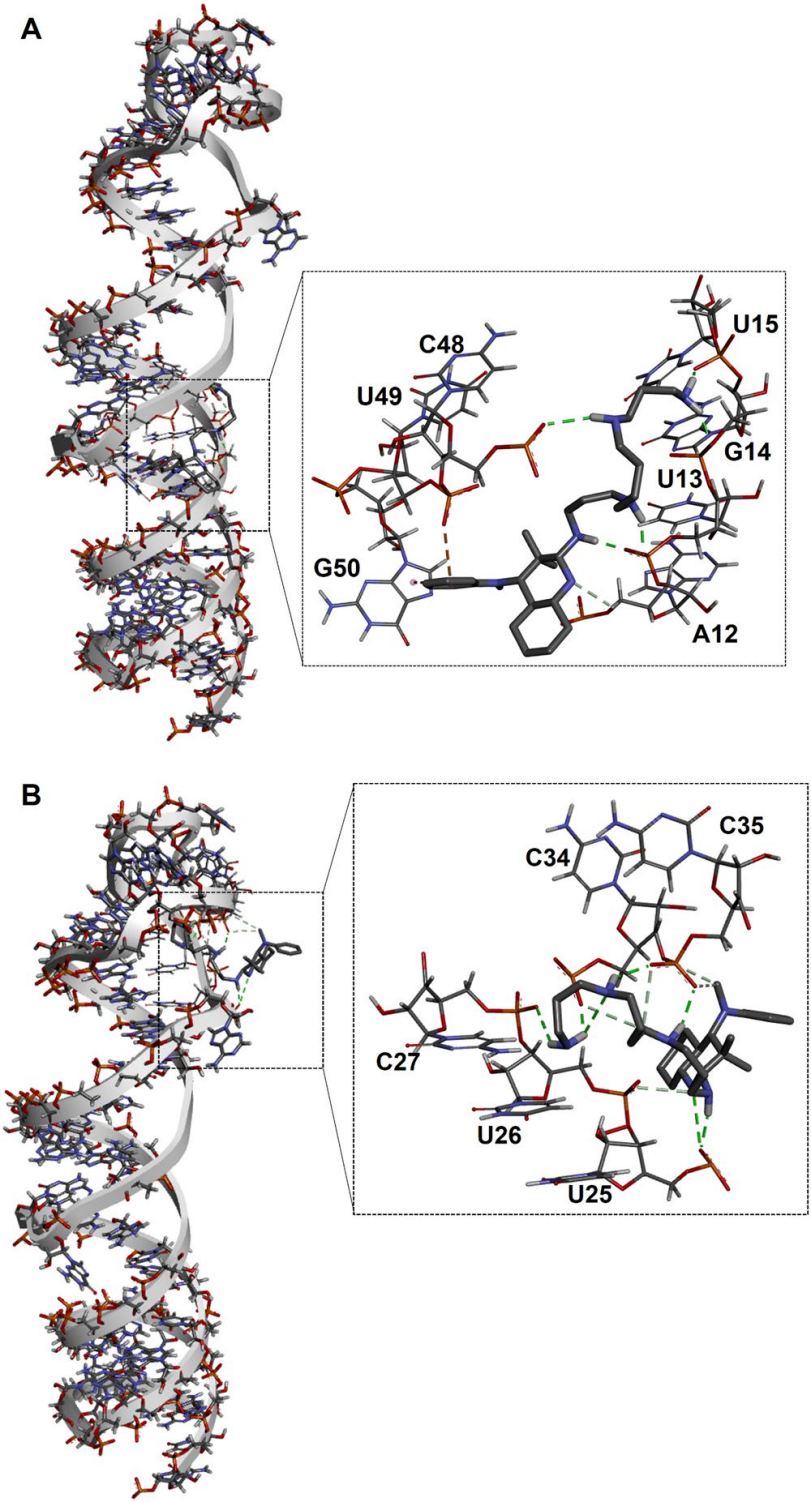
Docking of compound **PA-1** with the pre-miR-372 hairpin loop performed using autodock 4 where the grid boxes were fixed on the entire RNA sequence. (**A**) First possible docking of compound 1 close to the G16 internal bulge and (**B**) second possible docking conformation of compound **PA-1** at the junction between the stem and the terminal loop.

We then performed footprinting experiments on pre-miR-372, whose primary and secondary structures are illustrated in Fig. 6A, in the absence and in the presence of increasing concentrations of **PA-1**. Digestion of pre-miR-372 with lead(II) acetate in the presence of **PA-1** shows a clear footprint in the stem region between G16 and C20 as well as in the loop region between U28 and A36 (Fig. 6B, lanes 3–8 and Supplementary Fig. S8), while bands corresponding to A11-U15 and A44 are enhanced by increasing concentrations of **PA-1**. According to these results, digestion of pre-miR-372 using Dicer enzyme shows that **PA-1** induced a global inhibition of Dicer cleavage that is particularly marked for residues U15-G19, A24-C27 and A42 (Fig. 6C, lanes 5–7 and Supplementary Fig. S9). Digestion with RNase S1 on single-stranded RNA residues, confirms an interaction of **PA-1** with the loop region G29-U33 (Fig. 6D, lanes 5–8 and Supplementary Fig. S9). These results are supported by molecular docking studies^41^ allowing the visualization of the specific interactions between **PA-1** and pre-miR-372 (Fig. 7), which revealed that **PA-1** could bear two possible binding sites: (i) one in the stem region near the bulge at sites A12-U15 and C48-G50 (Fig. 7A) and (ii) the other one at the junction between the stem and the terminal loop at sites C25-C27 and C34-C35 (Fig. 7B). In both cases, hydrogen bonds between both the polyamine side chain and the nitrogen atoms of the aromatic moiety with the RNA phosphates dominate the interaction. Hydrophobic interactions (π-π interactions) between the terminal phenyl group of the aromatic moiety and RNA nucleobases also reinforce binding. Noteworthy, even if **PA-1** can bind to two different sites, it seems that it does not bind both simultaneously since the stoichiometry of the interaction fits the 1:1 model. These observations, together with the miRnome and RT-PCR quantification of affected miRNAs showed in previous sections, suggest that in principle, **PA-1** is susceptible to interact with other structured pre-miRNAs or pri-miRNAs. We thus preformed molecular docking studies on pre-miR-373, pre-miR-22, pre-miR-15a and pre-miR-31. The obtained results showed that the interaction of **PA-1** occurs on these RNA fragments always close to an internal bulges involving a purine residue and inducing a distortion of the double helix and the creation of an appropriate binding pocket (Supplementary Fig. S10). This implies that **PA-1** is not able to discriminate among miRNAs precursors containing this kind of motives. However, the experimental results presented here show that **PA-1** does not affect the entire miRnome but only a small set of oncogenic miRNAs. Indeed, it has been already demonstrated that a number of factors influence the intracellular effects on targeted miRNAs. First of all, binding of miRNA inhibitors must occur at functional sites, i.e. Drosha or Dicer processing site, in order to induce the inhibition of miRNA processing^25,42^. Furthermore, even if a small molecule is able to bind and efficiently inhibit particular miRNA precursors, levels of miRNA expression could be too low to induce an intracellular effect and only highly expressed miRNA could be druggable targets^42^.

In conclusion, our study highlighted that functionalized polyamines could function as inhibitors of miRNA biogenesis *in vitro* and in cancer cells upon binding to miRNA precursors. The most active molecule, **PA-1**, was able to bind pre-miR-372 and to inhibit its processing *in vitro*. A dose-dependent and specific inhibition of AGS proliferation concomitantly to a decrease in miR-372 amount and derepression of LATS2 expression suggested a very promising future for functionalized polyamines as selective miRNAs interfering agents. In fact, even if **PA-1** lacks specificity for a particular miRNA, it affects a restricted network of miRNAs and maintains a specific inhibitory effect on cancer cells proliferation. Importantly, **PA-1** also inhibits proliferation of cancer stem cells. These latter constitute the most aggressive and therapy-resistant cancer cells population and the inhibition of their proliferation interfering with their altered miRNA network has already been identified as a particularly promising anticancer approach. Altogether, the obtained results highlight the potential of polyamines in miRNAs-based approaches for cancer treatment and open the way for future medicinal chemistry studies for this class of compounds.

## Methods

### RNA and biochemicals

All buffers and solutions employed in FRET assays, K_D_ measurements and footprint experiments were filtered through 0.22 μm Millipore filters (GP ExpressPLUS membrane).

RNA and DNA oligonucleotides^18,19^ were purchased from IBA GmbH (Goettingen, Germany). A mixture of yeast pre- and mature tRNAs (containing > 30 different species) was purchased from Sigma (type X-SA).

RNase T1 was purchased from Ambion (1000U/µL), while nuclease S1 from Promega (89U/µL). Human recombinant Dicer enzyme (Genlantis) is at 0.5U/µL. Tris·HCl 20 mM, pH 7.4 containing 12 mM NaCl, 3 mM MgCl_2_ and 1 mM DTT was used for FRET assays, K_D_ determination and RNases footprint experiments (Buffer A). Tris·acetate 50 mM, pH 7.4 containing 12 mM NaCl and 3 mM MgCl_2_ was employed for lead(II) footprint experiments.

### Fluorescence-based assays

The FRET-Dicer assay and K_D_ determinations were performed in 384-well black plates (Greiner bio-one) in a final volume of 40 µL using a 5070 EpMotion automated pipetting system (Eppendorf). Each experiment was performed in duplicate and repeated three times. Refolding of the RNA was performed using a thermocycler (ThermoStatPlus Eppendorf) as follows: the RNA (5′-FAM-pre-miR-372-3′-DAB for FRET experiments and 5′-FAM-pre-miR-372 for K_D_ measurements), diluted in 1 mL of buffer A, was first denatured by heating to 90 °C for 2 min and then cooled to 4 °C for 10 min, followed by incubation at 25 °C for 15 min. After refolding, the RNA was diluted to a working concentration of 100 nM (for FRET-Dicer assays) or 10 nM (for binding assays) through addition of the appropriate amount of buffer A. During FRET-Dicer assay, 20 µL of pre-miR-372 beacon solution were added in each well containing 10 µL of each desired ligand concentration. These reaction mixtures were pre-incubated at room temperature 30 min. 0.25 U of human recombinant Dicer (Genlantis) in 10 µL were then added (final volume 40 µL) and the fluorescence increase measured every 15 minutes for 4 h (screening assay) or after 4 h (IC_50_ measurements). For IC_50_ experiments, each ligand was added in 12 dilutions (from 0.244 µM to 500 µM) and the fluorescence increase measured after 4 hours. During binding assays, 30 µL of pre-miR-372 beacon solution were added in each well containing 30 µL of each desired ligand concentration (final volume 60 µL). Each ligand was added in 15 dilutions (from 61 nM to 1 mM) and fluorescence was measured after incubating the plate at 4 °C overnight. The fluorescence was measured on a GeniosPro (Tecan) with an excitation filter of 485 ± 10 nm and an emission filter of 535 ± 15 nm. Each point was measured 10 times with a 500 μs integration time and averaged. Inhibition data were analyzed using Graphpad Prism 5 software using a nonlinear regression following the equation: Y = Bottom + (Top−Bottom)/1 + 10^[(LogIC50-X) × Hills Slope]^). Binding profiles were well modeled using a simple model assuming a one to one stoichiometry. In competition experiments the RNA beacon was first mixed with 100eq. of tRNA mix or DNA duplex.

### RNase and lead(II) footprinting assays

0.2 nmol of 5′-FAM-pre-miR-372 were diluted in 100 µL of buffer A (for RNase footprints) or buffer B (for lead(II) footprints) (final concentration 1 nM), were first denatured by heating to 90 °C for 2 min and then cooled to 4 °C for 10 min, followed by incubation at 25 °C for 15 min. One microliter of RNase T1 (final concentration 1 U/µL) and nuclease S1 (final concentration 89 U/µL) (diluted in buffer A) were added to the RNA beacon preincubated in the absence or in the presence of compound **PA-1** at 4 °C overnight (final reaction volume 10 µL). The reaction was incubated 15 min at 37 °C and stopped by ethanol precipitation (100 µL). Two microliters of lead(II) acetate (40 mM in buffer B) were added to the RNA beacon preincubated in the absence or in the presence of compound **PA-1** at 4 °C overnight (final reaction volume 10 µL) and incubated 5 min at 37 °C, then precipitated with ethanol (100 µL). Two microliters of human recombinant Dicer (0.5U/µL, Genlantis) were added to the RNA beacon preincubated in the absence or in the presence of compound **PA-1** at 4 °C overnight (final reaction volume 10 µL). The reaction was incubated 4 h at 37 °C and stopped by ethanol precipitation (100 µL). All footprinting samples were resuspended in 95% formamide and heated at 90 °C for 2 min before being loaded onto a denaturing 20% polyacrylamide (19:1 acrylamide:bisacrylamide) containing 7.5 M urea in 1X TBE buffer (50 mM Tris base, 55 mM boric acid, 1 mM EDTA). To identify RNase and lead(II) footprints. Gels were scanned with a Versadoc (Biorad) at 480 nm.

### Cell culture and treatment

All the tissue culture reagents were from Invitrogen (France). AGS cells (ATCC CRL 1739) and NCI-N87 (ATCC CRL 5822) cells were routinely grown in DMEM/F12 medium, whereas MKN74 (JCRB0255), MKN28 (JCRB0253), MKN7 (JCRB1025) cells were grown in RPMI 1640 medium. Both media were supplemented with 200 mM L-glutamine and 10% heat-inactivated fetal calf serum (FCS). Cells were cultured at 37 °C in a 5% CO_2_ atmosphere. Before being added directly in the culture media at the final concentration indicated in the figures, the conjugates were diluted in serum-free medium from a 50 mM stock solution in sterile water. The culture medium, with or without the conjugates, was changed every other day.

### Cell viability

Cells were plated in a 96-well microplate at 1,500 cells per well. The compounds were added the following day at the indicated concentrations. Cell viability was measured using the CellTiter96 AQueous One Solution Reagent (Promega), as recommended. Absorbance at 492 nm was read on a Bio-Tek spectrophotometric microplate reader.

### Quantitative RT-PCR

Total RNA was extracted using Trizol reagent (Invitrogen), according to the manufacturer’s protocol. RNA concentrations and quality were determined on a NanoDrop spectrophotometer (NanoDrop Technologies) and Bioanalyzer (Agilent), respectively.

TaqMan® microRNA, U6 snRNA and SNORD49A assays (Applied Biosystems) were used to quantify the expression of mature miRNA according to the manufacturer’s instructions and our previous reports^18,19,32^. No DNase treatment was performed for miRNA analysis. Specific miRNA-primed reverse transcription was carried out on 20 ng of total RNA using the TaqMan® MicroRNA Reverse Transcription Kit (Applied Biosystems) in a final volume of 7.5 μl, with the following steps: 30 min at 16 °C, 30 min at 42 °C, 5 min at 85 °C and a pause at 4 °C. Real time qPCR was performed in duplicate 15 μl per reaction on 1/20th diluted cDNA mixed to the 20X specific TaqMan® miRNA real time primer/probe and the 2X TaqMan® Universal PCR master mix, with the following steps: 20 s at 95 °C, [1 s at 95 °C, 20 s at 60 °C] for 40 cycles. MiRNAs levels were normalized to both U6 snRNA and SNORD49A.

The pri-miR-371-372-373 and LATS2 mRNA levels were determined by RT-qPCR; cDNA was synthesized from 500 ng total RNA with the Maxima First Strand cDNA Synthesis kit including a DNAse treatment (Thermo Scientific) in a 10 μl, as recommended by the manufacturer. Real time qPCR was performed on 1/100th diluted cDNA reaction, 2X GoTaq® qPCR Master mix (Promega), and 0.5 μM validated, specific primers (pri-miR and LATS2 primers are described in^32^ and housekeeping genes HPRT1 and TBP primers in^42^). The pri-miR and LATS2 levels were normalized to both HRPT1 and TBP. Real time qPCRs for both miRNA and pri-miRNA were performed on a RotorGene cycler (Qiagen). Relative expressions were calculated using the comparative Ct method.

### Immunofluorescence

Cells grown on glass coverslips were fixed with 3% paraformaldehyde in phosphate-buffered saline and treated with the anti-LATS2 antibody (Bethyl) as described^18,19^.

### Transient transfections of oligonucleotides and luciferase reporter systems

Cells were plated at 5.10^4^ cells/well in a 24-well plate. Transfections were performed using Lipofectamine 2000 (Invitrogen) according to the manufacturer’s protocol. Antisense oligonucleotides were transfected as described in^30^. For reporter assays, cells were treated with compound PA-**1** before being transfected the following day with 100 ng/well of either pGL4-PM372 or pGL4-mut372 firefly luciferase reporter^40^, mixed with 10 ng/well pRL-SV40 *Renilla* luciferase vector (Promega), used as a control for transfection efficiency. Firefly and *Renilla* luciferase activities were measured 48 h post-transfection using the Dual Luciferase Assay (Promega). Firefly luciferase activities were normalized for transfection efficiency by *Renilla* luciferase activity.

### Tumorsphere culture and treatment

GC06 and GC10 are patient-derived gastric adenocarcinoma xenografts, which were successfully established by serial transplantation in immunocompromized mice as previously described^39,43^. Histopathological characteristics were confirmed to be similar to those of the primary tumor after histological analysis of the tumor xenografts processed following standard procedures.

Dissociated GC cells (1000/well, n = 10) were seeded in non-adherent 96-well culture plates, previously coated with a 10% poly-2-hydroxyethyl methacrylate (Sigma) solution in 95% (v/v) ethanol and dried overnight at 56 °C in serum-free GlutaMAX-DMEM/F12 medium supplemented with 20 ng/mL of EGF, 20 ng/mL of basic-FGF, N-2, 0.3% glucose, 5 µg/mL of insulin, 50 IU/mL of penicillin/streptomycin (from Invitrogen and Sigma) and cultured at 37 °C in a humidified 5% CO_2_ atmosphere. The effects of **PA-1** were evaluated after 5 days of treatment counting the number of tumorspheres per well under a Zeiss inverted light microscope equipped with a 10X objective or measuring cell viability if the tumorpsheres were ambiguous to discriminate.

### Statistical analysis

Statistical significance was calculated using the One-Way analysis of variance (ANOVA) for multiple comparisons on GraphPad Prism, version 7.02 (La Jolla, CA, USA). Differences were considered significant at p < 0.05.

## Electronic supplementary material

Supplementary Information

## References

[1] Kim VN, Han J, Siomi MC (2009). Biogenesis of small RNAs in animals. Nat. Rev.Mol. Cell Biol..

[2] Ambros V (2008). The evolution of our thinking about microRNAs. Nat. Med..

[3] Wilson RC, Doudna JA (2013). Molecular mechanisms of RNA interference. Annu. Rev. Biophys..

[4] MacRae IJ, Ma E, Zhou M, Robinson CV, Doudna JA (2008). *In vitro* reconstitution of the human RISC-loading complex. Proc. Natl Acad. Sci. USA.

[5] Iorio MV, Croce C (2012). M. microRNA involvement in human cancer. Carcinogenesis.

[6] Li Z, Rana TM (2014). Therapeutic targeting of microRNAs: current status and future challenges. Nat. Rev. Drug Discov..

[7] Ling H, Fabbri M, Calin GA (2013). MicroRNAs and other non-coding RNAs as targets for anticancer drug development. Nat. Rev. Drug Discov..

[8] Di Giorgio A, Tran TPA, Duca M (2016). Small-molecule approaches toward the targeting of oncogenic microRNAs: roadmap for the discovery of RNA modulators. Future Med. Chem..

[9] Velagapudi SP, Vummidi BR, Disney MD (2015). Small molecule chemical probes of microRNA function. Curr. Opin. Chem. Biol..

[10] Gumireddy K (2008). Small-molecule inhibitors of microrna miR-21 function. Angew. Chem. Int. Ed. Engl..

[11] Velagapudi SP, Disney MD (2014). Two-dimensional combinatorial screening enables the bottom-up design of a microRNA-10b inhibitor. Chem. Commun. (Camb).

[12] Velagapudi SP (2017). Defining RNA-small molecule affinity landscapes enables design of a small molecule inhibitor of an oncogenic non-coding RNA. ACS Central Science.

[13] Shan G (2008). A small molecule enhances RNA interference and promotes microRNA processing. Nat. Biotechnol..

[14] Abell NS, Mercado M, Cañeque T, Rodriguez R, Xhemalce B (2017). Click Quantitative Mass Spectrometry Identifies PIWIL3 as a Mechanistic Target of RNA Interference Activator Enoxacin in Cancer Cells. J. Am. Chem. Soc..

[15] Shi Z (2013). AC1MMYR2, an inhibitor of dicer-mediated biogenesis of Oncomir miR-21, reverses epithelial-mesenchymal transition and suppresses tumor growth and progression. Cancer Res..

[16] Ren Y (2015). AC1MMYR2 impairs high dose paclitaxel-induced tumor metastasis by targeting miR-21/CDK5 axis. Cancer Lett..

[17] Ren Y (2016). Reprogramming carcinoma associated fibroblasts by AC1MMYR2 impedes tumor metastasis and improves chemotherapy efficacy. Cancer Lett..

[18] Vo DD (2014). Targeting the production of oncogenic microRNAs with multimodal synthetic small molecules. ACS Chem. Biol..

[19] Vo DD (2016). Oncogenic microRNAs biogenesis as a drug target: structure-activity relationship studies on novel aminoglycoside conjugates. Chem. Eur. J..

[20] Cho WJ (2009). S. miR-372 regulates cell cycle and apoptosis of ags human gastric cancer cell line through direct regulation of LATS2. Mol. Cells.

[21] Voorhoeve PM (2006). A genetic screen implicates miRNA-372 and miRNA-373 as oncogenes in testicular germ cell tumors. Cell.

[22] Connelly CM, Moon MH, Schneekloth JS (2016). The Emerging Role of RNA as a Therapeutic Target for Small Molecules. Cell Chem. Biol..

[23] Connelly CM, Boer RE, Moon MH, Gareiss P, Schneekloth JS (2017). Discovery of Inhibitors of MicroRNA-21 Processing Using Small Molecule Microarrays. ACS Chem. Biol..

[24] Velagapudi SP, Gallo SM, Disney MD (2014). Sequence-based design of bioactive small molecules that target precursor microRNAs. Nat. Chem. Biol..

[25] Tran TPA, Vo DD, Di Giorgio A, Duca M (2015). Ribosome-targeting antibiotics as inhibitors of oncogenic microRNAs biogenesis: Old scaffolds for new perspectives in RNA targeting. Bioorg. Med. Chem..

[26] Delcros JG (2006). Effect of polyamine homologation on the transport and biological properties of heterocyclic amidines. J. Med. Chem..

[27] Wang C, Delcros JG, Biggerstaff J, Phanstiel O (2003). Molecular requirements for targeting the polyamine transport system. Synthesis and biological evaluation of polyamine-anthracene conjugates. J. Med. Chem..

[28] Tomasi S (2006). Solid-phase synthesis of polyfunctionalized natural products: application to usnic acid, a bioactive lichen compound. J. Comb. Chem..

[29] Agostinelli E (2010). Polyamines: fundamental characters in chemistry and biology. Amino Acids.

[30] Mitchell JL, Thane TK, Sequeira JM, Thokala R (2007). Unusual aspects of the polyamine transport system affect the design of strategies for use of polyamine analogues in chemotherapy. Biochem. Soc. Trans..

[31] Lightfoot HL, Hall J (2014). Endogenous polyamine function-the RNAperspective. Nucleic Acid Res..

[32] Belair C (2011). Helicobacter pylori interferes with an embryonic stem cell micro RNA cluster to block cell cycle progression. Silence.

[33] Martin M (2011). Cutadapt removes adapter sequences from high-throughput sequencing reads. EMBnet Journal.

[34] An J, Lai J, Lehman ML, Nelson C (2013). C. miRDeep*: an integrated application tool for miRNA identification from RNA sequencing data. Nucleic Acids Res..

[35] Liu X (2010). MicroRNA-31 functions as an oncogenic microRNA in mouse and human lung cancer cells by repressing specific tumor suppressors. J. Clin. Invest..

[36] Wong N, Wang X (2015). miRDB: an online resource for microRNA target prediction and functional annotations. Nucleic Acids Res..

[37] Agarwal V, Bell GW, Nam JW, Bartel DP (2015). Predicting effective microRNA target sites in mammalian mRNAs. Elife.

[38] Clevers H (2011). The cancer stem cell: Premises, promises and challenges. Nat. Med..

[39] Nguyen PH (2017). Characterization of Biomarkers of Tumorigenic and Chemoresistant Cancer Stem Cells in Human Gastric Carcinoma. Clin. Cancer Res..

[40] Staedel C (2015). Inhibition of Gastric Tumor Cell Growth Using Seed-targeting LNA as Specific, Long-lasting MicroRNA Inhibitors. Mol. Ther. Nucleic Acids.

[41] Morris GM (2009). AutoDock4 and AutoDockTools4: Automated docking with selective receptor flexibility. J. Comp. Chem..

[42] Costales MG (2017). Small molecule inhibition of microRNA-210 reprograms an oncogenic hypoxic circuit. J. Am. Chem. Soc..

[43] Nguyen PH (2016). All-trans retinoic acid targets gastric cancer stem cells and inhibits patient-derived gastric carcinoma tumor growth. Oncogene.

