# Supplementary material for: Modulation of oncogenic miRNA biogenesis using functionalized polyamines
Source: Sci Rep. 2018 Jan 26;8:1667. doi: 10.1038/s41598-018-20053-5 (PMC5786041; doi:10.1038/s41598-018-20053-5)
Supplement: Supplementary file 1 — Supplementary Information [file 41598_2018_20053_MOESM1_ESM.pdf]

SUPPLEMENTARY INFORMATION

Modulation of oncogenic miRNA biogenesis using functionalized polyamines

Cathy Staedel[1,*], Thi Phuong Anh Tran[2], Julie Giraud[3], Fabien Darfeuille[1], Audrey Di Giorgio[2], Nicolas J. Tourasse[1], Franck Salin[4], Philippe Uriac[5], and Maria Duca[2*]

[1]Université de Bordeaux, INSERM, ARNA, 33076 Bordeaux, France

[2]Université Côte d'Azur, CNRS, ICN, 06107 Nice, France

[3]Université de Bordeaux, INSERM, BARITON, 33076 Bordeaux, France

[4]Université de Bordeaux, INRA, BIOGECO, 33612 Cestas, France

[5]Université de Rennes 1, CNRS, ISCR, 35042 Rennes, France

[*] To whom correspondence should be addressed: Tel: +33 4 92076153; Fax: +33 4 92076151; Email: maria.duca@unice.fr. Correspondence may also be addressed to Cathy Staedel: Tel: +33 5 57 57 45 65; Fax: +33 5 57 57 10 15; Email: cathy.staedel@inserm.fr

SUPPLEMENTARY METHODS

MiRnome. Cells were plated in 6-well plates and treated or not with 25 µM of **PA-1** for 4 days during their exponential growth phase, in triplicates. Cells were lyzed and RNA was extracted using the mirVana™ miRNA isolation lit (Ambion) according to the manufacturer's protocol. 100 fentomoles of spike-in cel-miR-39 (Qiagen) were added to each sample during the lysis step. The small RNA (<200 nt) fraction were enriched and purified by passage on two sequential glass-fiber filters, as recommended. Small RNAs were quantified and analyzed using the Agilent® RNA 6000 Nano chip on an Agilent® Bioanalyzer instrument. Small RNA libraries were constructed using the Ion Total RNA-SEq kit and Ion Xpress™ RNA-Seq Barcode kit (Life Technologies, Foster City, CA). Template preparation, emulsion PCR and Ion Sphere Particles enrichment were performed using the PGM™ Hi-Q™ OT2 kit (Life Technologies), according to the manufacturer's instructions. Sequencing was performed in an Ion PGM Sequencer using an Ion 314 Chip and Ion PGM™ Hi-Q™ Sequencing Kit (Life Technologies). The miRNA sequences were demultiplexed and trimmed by the Torrent Suite software (Life Technologies).

Sequence analysis

Reads were trimmed of IonTorrent adapters (A-key and P1-key) using cutadapt 1.1[21a] run with an error rate of 20% and a minimum overlap between read and adapter of 20 bases (options "-e 0.20 -O 20"). Identification of miRNAs and quantification of their expression levels were carried out by means of miRDeep* 37[21b] using assembly hg19 (GRCh37) of the human genome (which is part of miRDeep*) and default options, except for the minimum phred quality score which was lowered from 20 to 18 for some datasets that were of lower quality. The human miRNA set included in miRDeep* was from miRBase v19 (http://www.mirbase.org/). To quantify the amount of the cel-miR-39 miRNA of *Caenorhabditis elegans* that was included as a control, the *C. elegans* ce11 (WBcel235) genome assembly was downloaded from the Ensembl database release 84 (http://www.ensembl.org/) and installed into miRDeep*, which was run with the same parameters as above. The *C. elegans* miRNA set was downloaded from miRBase v21.

Molecular Modeling and Docking

The MC-Fold/MC-Sym pipeline ((http:// http://www.major.iric.ca/MC-Pipeline/) is a web-hosted service for RNA secondary and tertiary structure prediction. The pipeline consists in uploading RNA sequence to MC-Fold, which output secondary structures that are directly input to MC-Sym, which outputs tertiary structures. Pre-miRNA sequences were obtained from the miRBase database (http://www.mirbase.org/). The hairpin loop of pre-miR-372 was chosen to predict the 3D structure using the MC-Fold/MC-Sym pipeline. Energy optimization was further conducted on the 3D model using the TINKER Molecular Modeling Package (http://dasher.wustl.edu/tinker/).

For docking with AutoDock,[41] polar hydrogen atoms, Kollman united charges and solvent parameters were applied to the RNA using pmol2q script (http://www.sourcefiles.org/Scientific/Biology/Proteins/pmol2q_2.3.0.tar.gz). This script converts the .pdb file format of the RNA template to the .pdbqt file format that is compatible with AutoDock program version 4 (http://autodock.scripps.edu/). Pre-miR-372/**PA-1** molecular docking was conducted using AutoDock program version 4. The rotational bonds of the ligand were treated as flexible, whereas the receptor was kept rigid. Grid box was fixed in order to include the entire RNA sequence. RNA-ligand interactions were analyzed and visualized using Discovery Studio Visualizer version 4.1 (http://accelrys.com/products/discovery-studio/).

Figure S1. A) Binding curves of compounds **PA-1-3** (concentrations from 61 nM to 1 mM) in the presence of pre-miR-372 and B) inhibition curves of compounds **1-3** incubated with pre-miR-372 in the presence of Dicer.

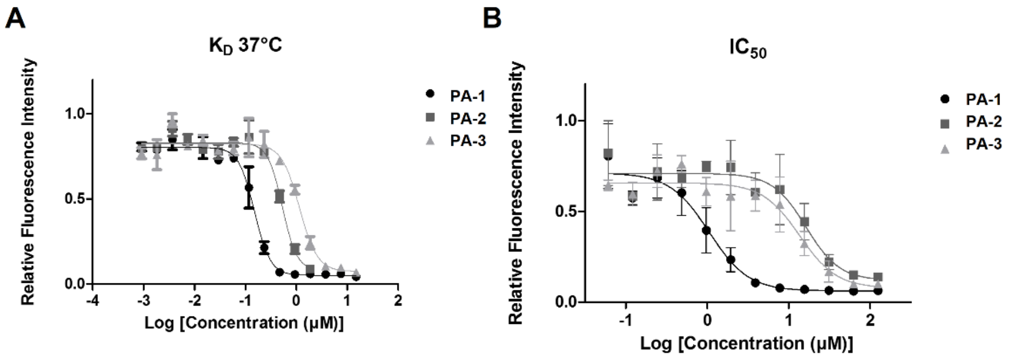

Figure S2. A) A) Relative growth rate of the gastric adenocarcinoma AGS, MKN74, MKN7, MKN28 and NCI-N87 cell lines in the absence (control) and in the presence of **PA-1** (25 µM). Growth was determined by (A492nm at day 4 - A492nm at day 2) / A492nm at day 2. Each point represents the mean ± standard deviation (SD) of growth rate data compared to those of untreated cells. B) Relative expression of relevant miRNAs in the different cell lines in basal growth conditions. Bars represent the means ± SD (n = 3) of RT-qPCR data of the miRNAs normalized to both RNU49 and snU6 and compared to AGS cells.

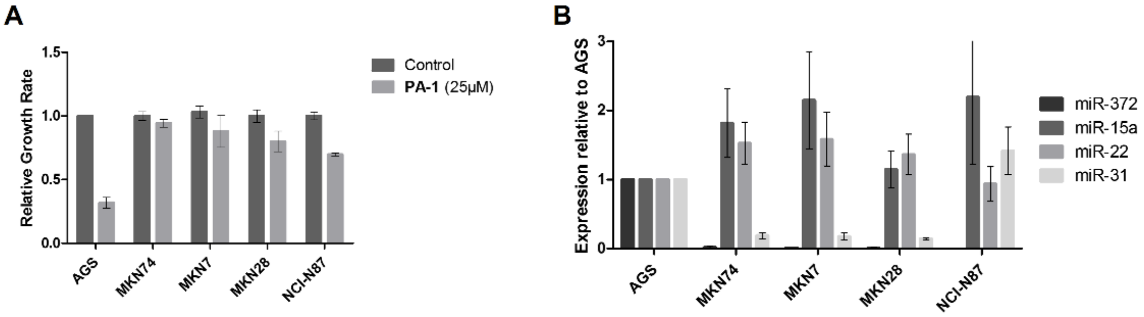

Figure S3. Relative growth rate of AGS cells (A) expressing high miR-372 levels and MKN74 cells (B) in the presence of spermine and putrescine. Growth was determined by (A_{492nm} at day 4 - A_{492nm} at day 2) / A_{492nm} at day 2. Each point represents the mean ± standard deviation (SD) of growth rate data compared to those of untreated cells.

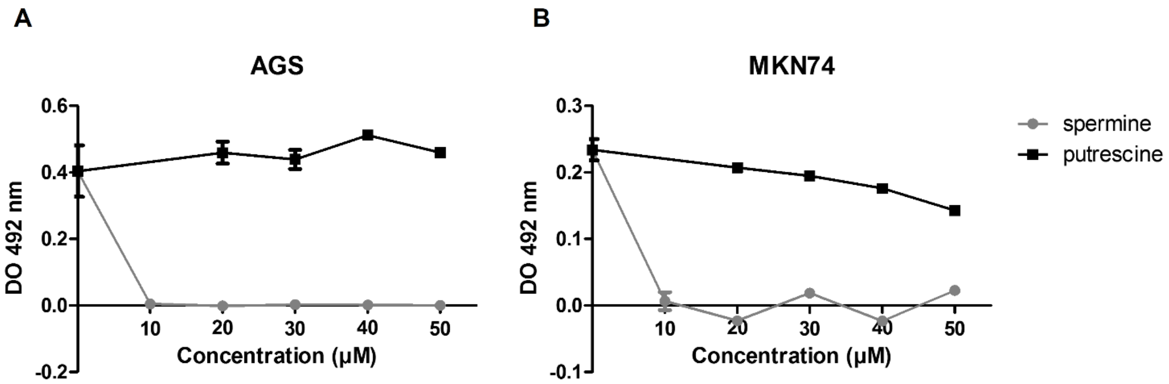

Figure S4. Cytostatic and reversible effect of **PA-1** on AGS cells. A) Cell cycle analysis by flow cyometry: upon 48h treatment with **PA-1** at the indicated concentrations, the cells were detached and fixed in 70% ethanol, as described in [32]; DNA was stained by propidium iodine (0.5 mg/ml in PBS, 0.1% BSA, 0.1% RNaseA). Representative DNA histograms are shown, with the percentage of cells in the different phases of the cell cycle (mean ± SD of quadruplicates); B) CDKN1A/p21 protein immunolabelling using fluorescence microscopy, in untreated cells and upon 24h or 72h treatment with **PA-1** at 25 μM. CDKN1A/p21 was labeled on paraformaldehyde-fixed cells using a mouse anti-human p21 monoclonal antibody (BD Pharmingen), followed by an Alexa 564-labeled anti-mouse antibody, and appears in red in the cell nucleus. Nuclei are revealed by Hoechst staining and appear in blue; C) RT-PCR quantification of CDKN1A/p21, PCNA and PDCD4 mRNAs (as described in [43]) after a 72 h treatment with increasing doses of **PA-1**. Bars represent the mean ± SD of the different gene expression normalized to the housekeeping genes and compared to untreated cells (n = 3); D) Cells were plated in 96-well microplates and treated with **PA-1** at 25 μM for 72h. Cell viability was assessed at this time point on a part of the wells (dark grey bars), while the others wells were rinsed twice with fresh growth medium and grown in the presence (medium grey bars) or in the absence (light grey bars) of **PA-1** (25 μM) for additional 48h. Cell viability was assessed at this later time point. Bars represent the absorbance at 492 nm of the CellTiter reagent metabolized by the viable cells (mean ± SD of quadruplicates).

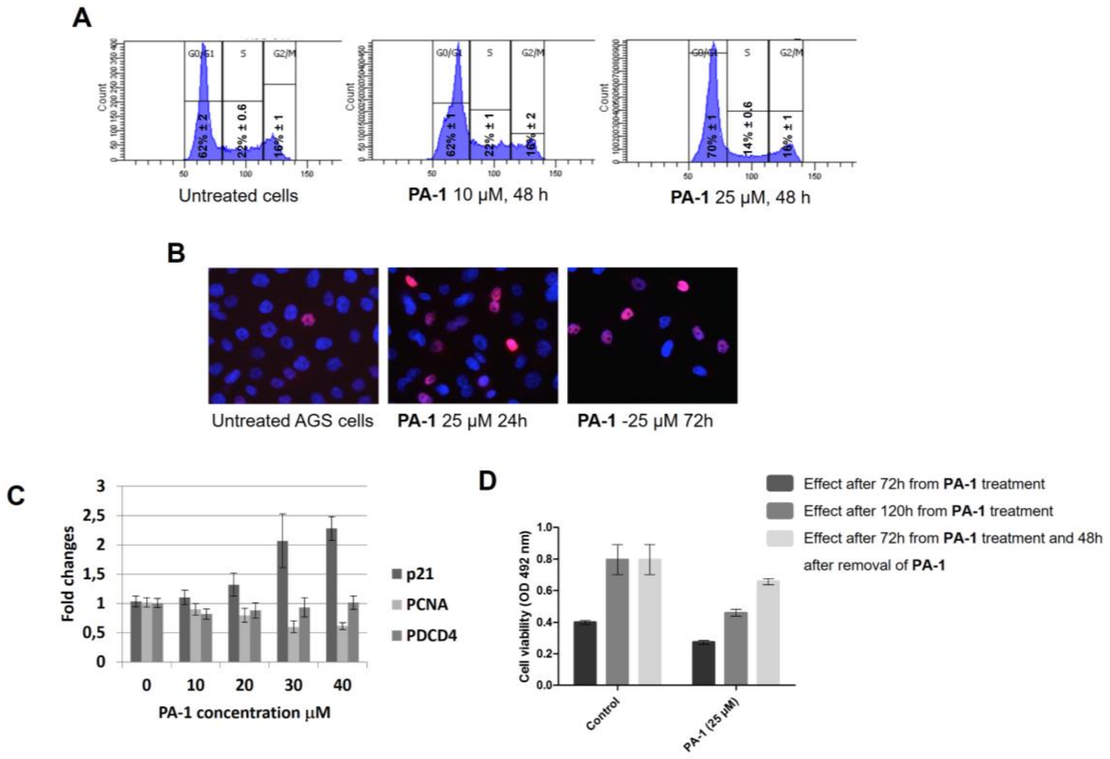

Figure S5. MiRNAs found to be downregulated (A) or upregulated (B) in the miRnome of AGS cells treated for 4 days with 25 µM of compound **PA-1,** compared to untreated cells. Bars represent the mean fold changes in the number of reads (% of total reads) for each miRNA, for triplicate samples, established for the AGS miRnome in Table S3.

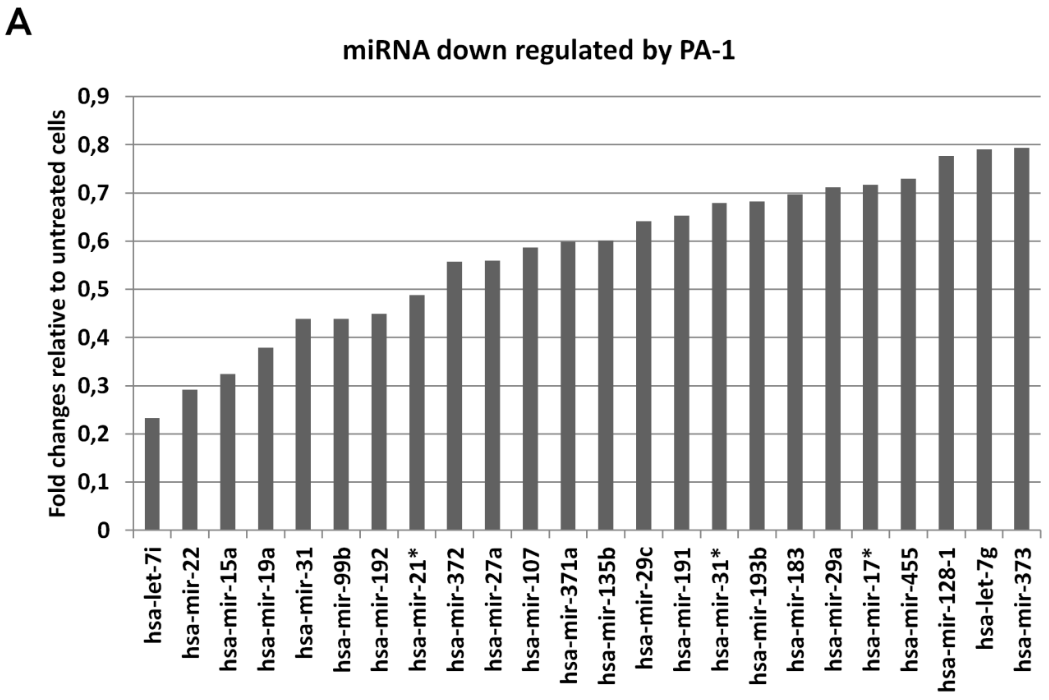

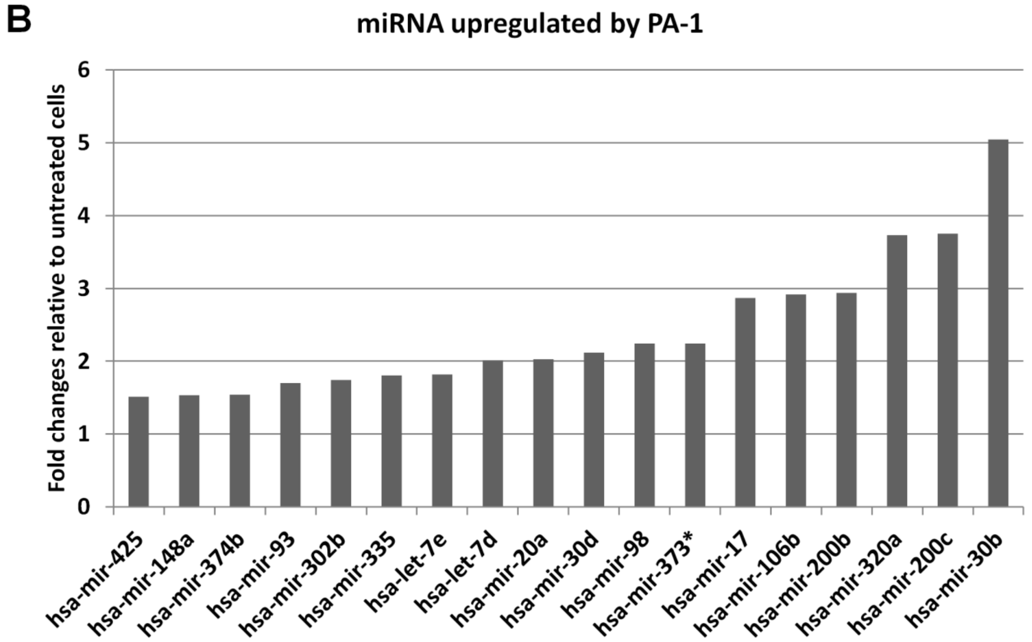

Figure S6. Verification by RTqPCR of the changes in the miRNA levels upon treatment with compound **PA-1**. The same triplicate samples as for the miRnome analysis were used. Bars are the mean ± SD of fold changes of miRNA in **PA-1**-treated AGS compared to untreated ones.

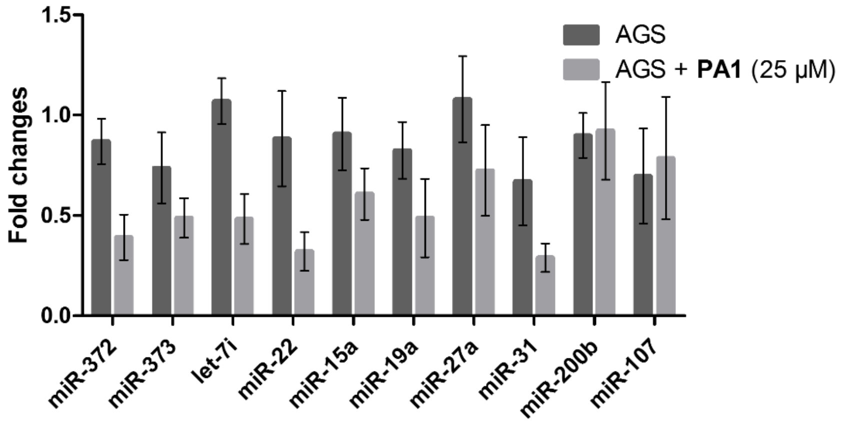

Figure S7. Inhibition of **PA-1**-mediated growth inhibition by complementation by a mimic of mature miR-372 (mim372). AGS were plated in 96-well plates at 2500 cells/well and treated with 25 μM **PA-1**. The following day, 0.1 or 1 μM mim372 or siControl double stranded oligoribonucleotides were transfected using Lipofectamin2000 (Invitrogen) as recommended and previously described[32]. Cell viability was measured 48h later. Bars represent the fold changes of absorbance at 492 nm relative to untreated siControl cells (mean ± SD in triplicates).

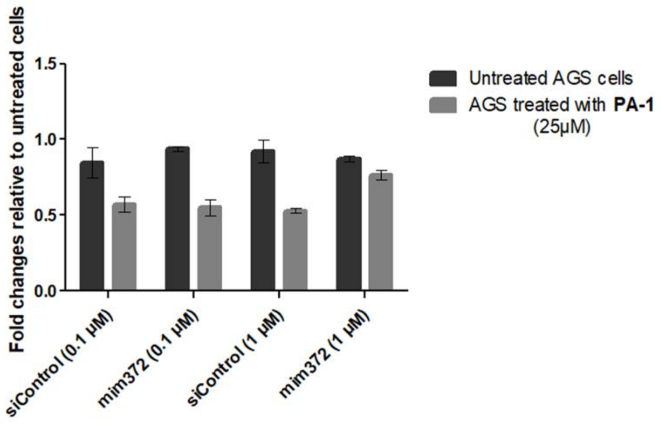

Figure S8. Quantification of lead(II) footprinting analysis relative to gel showed in Figure 2B.

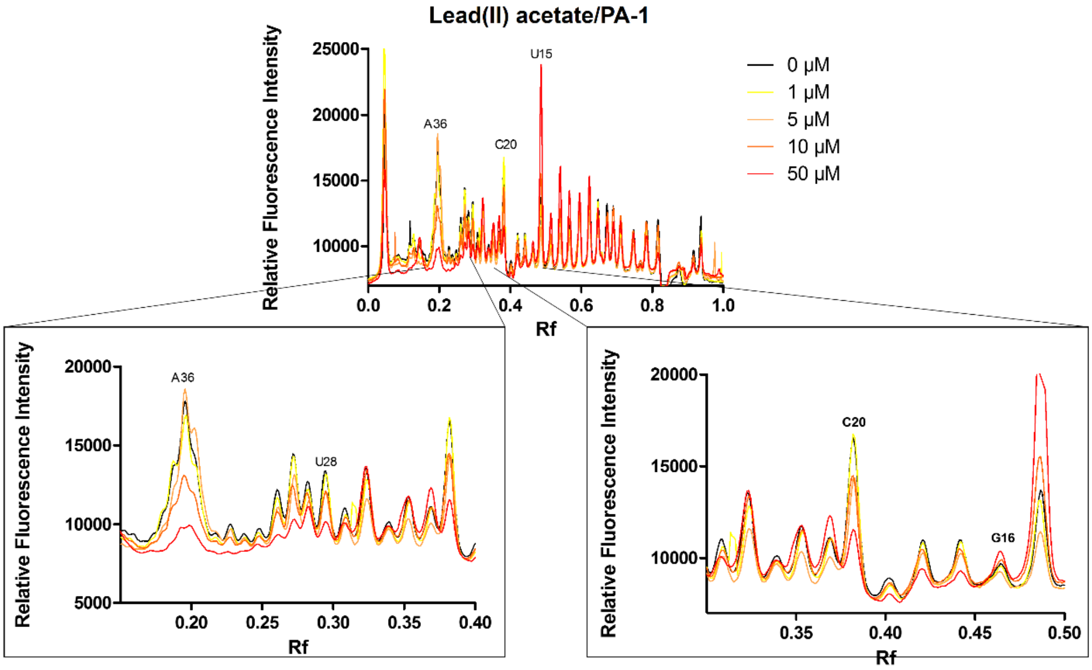

Figure S9. A) Quantification of Dicer footprinting analysis relative to gel showed in Figure 2C; B) Quantification of S1 RNase footprinting analysis relative to gel showed in Figure 2D.

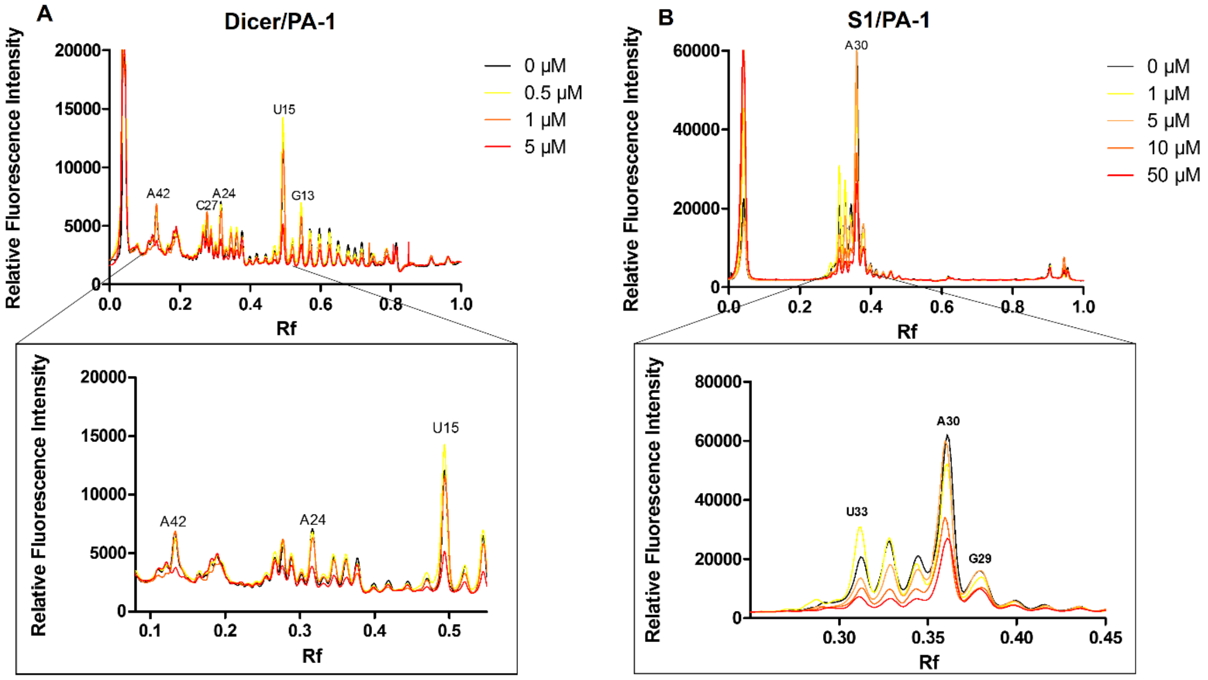

Figure S10. Docking of compound **PA-1** with the pre-miR-373 (A), pre-miR-22 (B) and pre-miR-31 (C) hairpin loops performed using autodock 4 where the grid boxes were fixed on the entire RNA sequence.

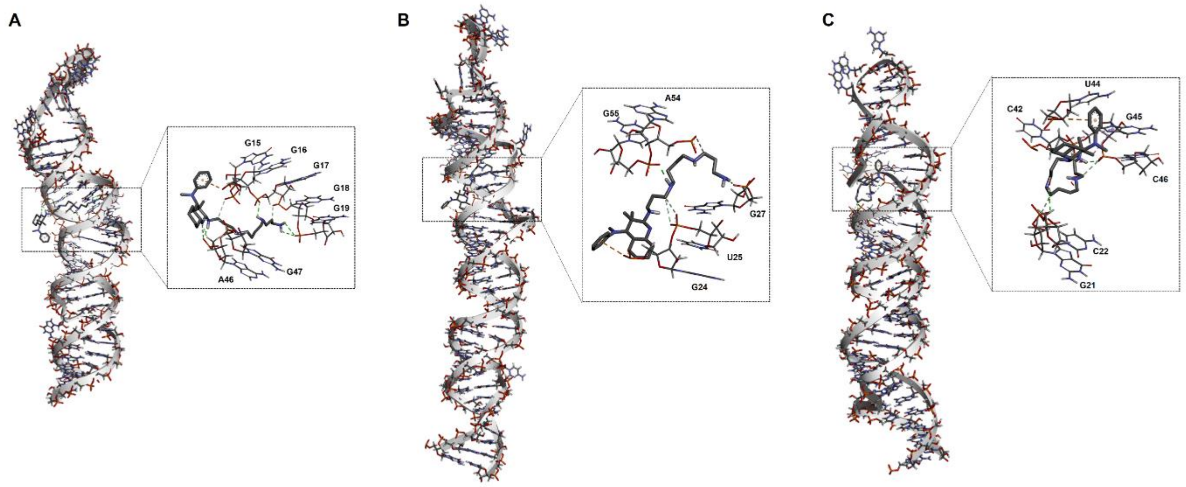

Figure S11. Uncropped gels corresponding to Figure 6 of the main manuscript. A) complete gel relative to Figure 6B with additional L and R control bands; B) complete gel relative to Figure 6C showing reproducibility of the result obtained in lanes 4-7 that have been repeated twice on the gel and C) complete gel relative to Figure 6D with additional increasing concentration of **PA-1** in the presence of S1 enzyme.

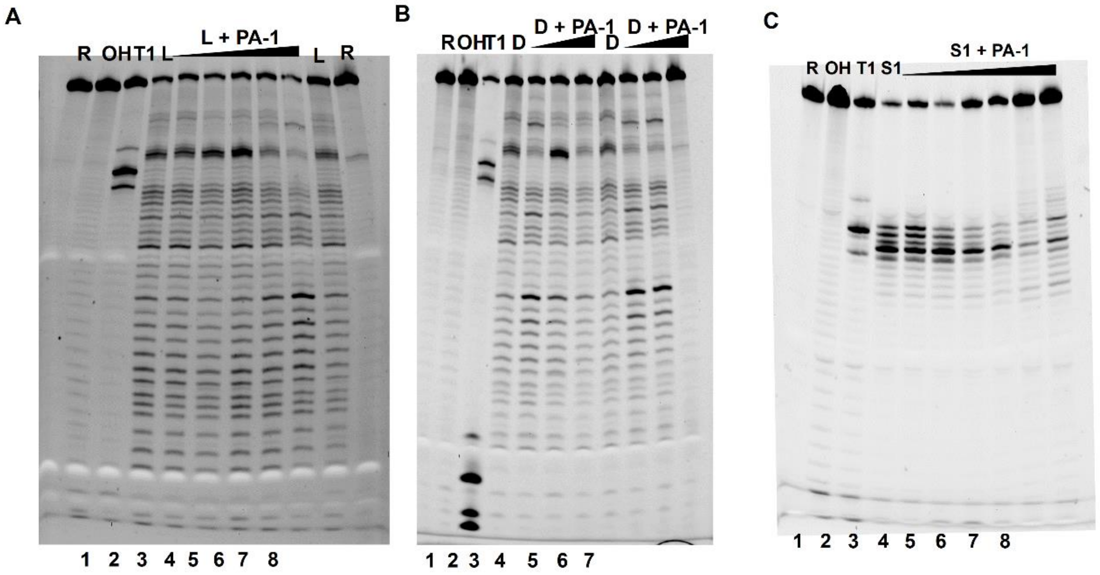

Table S1. MiRnome of untreated and PA-1-treated (4 days at 25 μM) AGS cells. Data are expressed as the percentage of each miRNA reads normalized to cel-miR-39 relative to the total number of miRNA reads of the sample (mean ± SD for the triplicate samples). The miRNAs are sorted in a decreasing order of their percent of total reads in the untreated AGS cells.

miR_ID	mature miR(highest read)	Untreated AGS		PA-1 treated AGS	
		Mean	*SD*	Mean	*SD*
hsa-mir-21	TAGCTTATCAGACTGATGTTGA(TAGCTTATCAGACTGATGTTGA)	16,781	*0,495*	20,314	*3,613*
hsa-mir-372	AAAGTGCTGCGACATTTGAGCGT(AAAGTGCTGCGACATTTGAGCGT)	16,746	*0,463*	9,329	*1,845*
hsa-mir-373	GAAGTGCTTCGATTTTGGGGTGT(GAAGTGCTTCGATTTTGGGGTGT)	15,798	*1,073*	12,545	*1,076*
hsa-mir-371a-5p	ACTCAAACTGTGGGGGCACT(ACTCAAACTGTGGGGGCACTT)	10,685	*0,664*	12,244	*1,554*
hsa-mir-31	AGGCAAGATGCTGGCATAGCT(AGGCAAGATGCTGGCATAGCTG)	3,795	*0,374*	1,664	*0,194*
hsa-let-7a	TGAGGTAGTAGGTTGTATAGTT(TGAGGTAGTAGGTTGTATAGTT)	2,355	*0,230*	2,836	*0,469*
hsa-mir-19a	TGTGCAAATCTATGCAAAACTGA(TGTGCAAATCTATGCAAAACTGA)	2,295	*0,131*	0,869	*0,009*
hsa-mir-23a	ATCACATTGCCAGGGATTTCC(ATCACATTGCCAGGGATTT)	2,051	*0,212*	2,200	*0,071*
hsa-mir-203a	GTGAAATGTTTAGGACCACTAG(GTGAAATGTTTAGGACCACTAG)	1,603	*0,130*	1,914	*0,165*
hsa-mir-23b	ATCACATTGCCAGGGATTACC(ATCACATTGCCAGGGATTA)	1,577	*0,122*	2,035	*0,184*
hsa-mir-99b	CACCCGTAGAACCGACCTTGCG(CACCCGTAGAACCGACCTTGCG)	1,522	*0,171*	0,668	*0,178*
hsa-mir-18a	TAAGGTGCATCTAGTGCAGATAG(TAAGGTGCATCTAGTGCAGATAG)	1,431	*0,165*	1,487	*0,285*
hsa-mir-371a-3p	AAGTGCCGCCATCTTTTGAGTGT(ACTCAAACTGTGGGGGCACTT)	1,354	*0,135*	0,810	*0,024*
hsa-let-7e	TGAGGTAGGAGGTTGTATAGTT(TGAGGTAGGAGGTTGTATAGTT)	1,288	*0,198*	2,342	*0,164*
hsa-mir-20a	TAAAGTGCTTATAGTGCAGGTAG(TAAAGTGCTTATAGTGCAGGTAG)	1,260	*0,073*	2,557	*0,058*
hsa-mir-27b	TTCACAGTGGCTAAGTTCTGC(TTCACAGTGGCTAAGTTCTG)	1,125	*0,105*	1,068	*0,103*
hsa-mir-191	CAACGGAATCCCAAAAGCAGCTG(CAACGGAATCCCAAAAGCAGCTG)	1,109	*0,055*	0,724	*0,056*
hsa-mir-125a	TCCCTGAGACCCTTTAACCTGTGA(TCCCTGAGACCCTTTAACCTGTG)	1,066	*0,073*	1,404	*0,192*
hsa-let-7g	TGAGGTAGTAGTTTGTACAGTT(TGAGGTAGTAGTTTGTACAGTT)	0,959	*0,146*	0,758	*0,098*
hsa-mir-27a	TTCACAGTGGCTAAGTTCCGC(TTCACAGTGGCTAAGTTCCG)	0,947	*0,095*	0,530	*0,073*
hsa-mir-29a	TAGCACCATCTGAAATCGGTTA(TAGCACCATCTGAAATCGGTTA)	0,705	*0,026*	0,502	*0,063*
hsa-let-7b	TGAGGTAGTAGGTTGTGTGGTT(TGAGGTAGTAGGTTGTGTGGTT)	0,678	*0,049*	0,709	*0,160*
hsa-mir-192	CTGACCTATGAATTGACAGCC(CTGACCTATGAATTGACAG)	0,582	*0,067*	0,261	*0,037*
hsa-mir-15b	TAGCAGCACATCATGGTTTACA(TAGCAGCACATCATGGTTTACA)	0,576	*0,070*	0,763	*0,068*
hsa-let-7i	TGAGGTAGTAGTTTGTGCTGTT(TGAGGTAGTAGTTTGTGCTGTT)	0,563	*0,004*	0,131	*0,031*
hsa-let-7d	AGAGGTAGTAGGTTGCATAGTT(AGAGGTAGTAGGTTGCATAGTT)	0,485	*0,033*	0,976	*0,087*
hsa-mir-200a	TAACACTGTCTGGTAACGATGT(TAACACTGTCTGGTAACGATGTT)	0,476	*0,060*	0,510	*0,080*
hsa-mir-34a	TGGCAGTGTCTTAGCTGGTTGT(TGGCAGTGTCTTAGCTGGTTGTT)	0,474	*0,047*	0,676	*0,047*
hsa-mir-660	TACCCATTGCATATCGGAGTTG(TACCCATTGCATATCGGAGTTGT)	0,443	*0,039*	0,570	*0,057*
hsa-mir-151a	TCGAGGAGCTCACAGTCTAGT(TCGAGGAGCTCACAGTCTAGTA)	0,416	*0,044*	0,570	*0,029*
hsa-mir-31	TGCTATGCCAACATATTGCCAT(AGGCAAGATGCTGGCATAGCTG)	0,406	*0,027*	0,276	*0,029*
hsa-mir-30d	TGTAAACATCCCCGACTGGAAG(TGTAAACATCCCCGACTGGAAG)	0,403	*0,034*	0,854	*0,082*
hsa-mir-93	CAAAGTGCTGTTCGTGCAGGTAG(CAAAGTGCTGTTCGTGCAGGT	0,385	*0,015*	0,655	*0,371*

	AG)				
hsa-mir-10a	TACCCTGTAGATCCGAATTTGTG(TACCCTGTAGATCCGAATTTGT)	0,381	*0,024*	0,359	*0,026*
hsa-mir-22	AAGCTGCCAGTTGAAGAACTGT(AAGCTGCCAGTTGAAGAACTGT)	0,372	*0,032*	0,108	*0,028*
hsa-mir-141	TAACACTGTCTGGTAAAGATGG(TAACACTGTCTGGTAAAGATGG)	0,356	*0,018*	0,373	*0,078*
hsa-mir-17	CAAAGTGCTTACAGTGCAGGTAG(CAAAGTGCTTACAGTGCAGGTAG)	0,350	*0,115*	1,003	*0,045*
hsa-mir-30b	TGTAAACATCCTACACTCAGCT(TGTAAACATCCTACACTCAGCT)	0,323	*0,049*	1,631	*0,203*
hsa-mir-200b	TAATACTGCCTGGTAATGATGA(TAATACTGCCTGGTAATGATGA)	0,318	*0,060*	0,935	*0,104*
hsa-mir-183	TATGGCACTGGTAGAATTCACT(TATGGCACTGGTAGAATTCACTG)	0,279	*0,061*	0,195	*0,027*
hsa-mir-15a	TAGCAGCACATAATGGTTTGTG(TAGCAGCACATAATGGTTTGTG)	0,261	*0,069*	0,085	*0,017*
hsa-mir-200c	TAATACTGCCGGGTAATGATGGA(TAATACTGCCGGGTAATGATGG)	0,259	*0,034*	0,971	*0,086*
hsa-mir-373*	ACTCAAAATGGGGGCGCTTTCC(GAAGTGCTTCGATTTTGGGGTGT)	0,253	*0,054*	0,568	*0,054*
hsa-mir-130a	CAGTGCAATGTTAAAAGGGCAT(CAGTGCAATGTTAAAAGGGCAT)	0,250	*0,047*	0,322	*0,011*
hsa-mir-106b	TAAAGTGCTGACAGTGCAGAT(TAAAGTGCTGACAGTGCAGATA)	0,232	*0,072*	0,677	*0,028*
hsa-mir-429	TAATACTGTCTGGTAAAACCGT(TAATACTGTCTGGTAAAACCGT)	0,229	*0,076*	0,220	*0,059*
hsa-mir-107	AGCAGCATTGTACAGGGCTATCA(AGCAGCATTGTACAGGGCTAT)	0,221	*0,028*	0,130	*0,013*
hsa-mir-455	GCAGTCCATGGGCATATACAC(GCAGTCCATGGGCATATACA)	0,187	*0,005*	0,137	*0,012*
hsa-mir-21*	CAACACCAGTCGATGGGCTGT(TAGCTTATCAGACTGATGTTGA)	0,178	*0,036*	0,087	*0,002*
hsa-mir-221	AGCTACATTGTCTGCTGGGTTTC(AGCTACATTGTCTGCTGGGTTT)	0,161	*0,039*	0,195	*0,005*
hsa-mir-374b	ATATAATACAACCTGCTAAGTG(ATATAATACAACCTGCTAAGTG)	0,160	*0,022*	0,246	*0,052*
hsa-mir-222	AGCTACATCTGGCTACTGGGT(AGCTACATCTGGCTACTGGGT)	0,140	*0,042*	0,162	*0,035*
hsa-mir-335	TCAAGAGCAATAACGAAAAATGT(TCAAGAGCAATAACGAAAAATGT)	0,137	*0,001*	0,247	*0,046*
hsa-mir-135b	TATGGCTTTTCATTCCTATGTGA(TATGGCTTTTCATTCCTATGTGA)	0,130	*0,009*	0,078	*0,014*
hsa-mir-98	TGAGGTAGTAAGTTGTATTGTT(TGAGGTAGTAAGTTGTATTGTT)	0,130	*0,005*	0,290	*0,303*
hsa-mir-7-1	CAACAAATCACAGTCTGCCATA(TGGAAGACTAGTGATTTTGTTGT)	0,121	*0,009*	0,169	*0,030*
hsa-mir-423	AGCTCGGTCTGAGGCCCCTCAGT(AGCTCGGTCTGAGGCCCCTCAGT)	0,119	*0,007*	0,136	*0,022*
hsa-mir-744	TGCGGGGCTAGGGCTAACAGCA(TGCGGGGCTAGGGCTAACAGCA)	0,116	*0,006*	0,162	*0,075*
hsa-mir-324	CGCATCCCCTAGGGCATTGGTGT(CGCATCCCCTAGGGCATTGGTGT)	0,114	*0,022*	0,164	*0,076*
hsa-mir-484	TCAGGCTCAGTCCCCTCCCGAT(TCAGGCTCAGTCCCCTCCCGAT)	0,106	*0,019*	0,091	*0,031*
hsa-mir-590	GAGCTTATTCATAAAAGTGCAG(GAGCTTATTCATAAAAGTGCAG)	0,098	*0,012*	0,135	*0,040*
hsa-mir-320a	AAAAGCTGGGTTGAGAGGGCGA(AAAAGCTGGGTTGAGAGGGCGA)	0,095	*0,014*	0,354	*0,070*
hsa-mir-128-1	TCACAGTGAACCGGTCTCTTT(TCACAGTGAACCGGTCTCTTT)	0,092	*0,002*	0,072	*0,012*
hsa-mir-193b	AACTGGCCCTCAAAGTCCCGCT(AACTGGCCCTCAAAGTCCCGCT)	0,091	*0,007*	0,062	*0,010*
hsa-mir-29c	TAGCACCATTTGAAATCGGTTA(TAGCACCATTTGAAATCGGTTA)	0,090	*0,000*	0,058	*0,008*
hsa-mir-423	TGAGGGGCAGAGAGCGAGACTTT(AGCTCGGTCTGAGGCCCCTCAGT)	0,090	*0,006*	0,081	*0,021*
hsa-mir-30e	TGTAAACATCCTTGACTGGAAG(TGTAAACATCCTTGACTGGAAG)	0,084	*0,011*	0,121	*0,026*
hsa-mir-185	TGGAGAGAAAGGCAGTTCCTGA(TGGAGAGAAAGGCAGTTCCTGA)	0,083	*0,010*	0,078	*0,019*
hsa-mir-17*	ACTGCAGTGAAGGCACTTGTAG(CAAAGTGCTTACAGTGCAGGTAG)	0,081	*0,000*	0,058	*0,006*
hsa-mir-532	CATGCCTTGAGTGTAGGACCGT(CATGCCTTGAGTGTAGGACCGT)	0,079	*0,030*	0,084	*0,023*

hsa-mir-148a	TCAGTGCACTACAGAACTTTGT(TCAGTGCACTACAGAACTTTGT)	0,078	*0,019*	0,120	*0,027*
hsa-mir-128-2	TCACAGTGAACCGGTCTCTTT(TCACAGTGAACCGGTCTCTTT)	0,077	*0,004*	0,068	*0,014*
hsa-mir-130b	CAGTGCAATGATGAAAGGGCAT(CAGTGCAATGATGAAAGGGCAT)	0,075	*0,034*	0,101	*0,021*
hsa-mir-301a	CAGTGCAATAGTATTGTCAAAGC(CAGTGCAATAGTATTGTCAAAG)	0,074	*0,018*	0,100	*0,010*
hsa-mir-302b	TAAGTGCTTCCATGTTTTAGTAG(TAAGTGCTTCCATGTTTTAGTAG)	0,071	*0,016*	0,123	*0,035*
hsa-mir-302d	TAAGTGCTTCCATGTTTGAGTGT(TAAGTGCTTCCATGTTTGAGTGT)	0,068	*0,027*	0,061	*0,015*
hsa-mir-365a	TAATGCCCCTAAAAATCCTTAT(TAATGCCCCTAAAAATCCTTAT)	0,067	*0,001*	0,094	*0,017*
hsa-mir-365b	TAATGCCCCTAAAAATCCTTAT(TAATGCCCCTAAAAATCCTTAT)	0,067	*0,001*	0,094	*0,017*
hsa-mir-935	CCAGTTACCGCTTCCGCTACCGC(CAGTTACCGCTTCCGCTACCG)	0,066	*0,025*	0,072	*0,018*
hsa-mir-425	AATGACACGATCACTCCCGTTGA(AATGACACGATCACTCCCGTTGA)	0,064	*0,015*	0,097	*0,025*
hsa-mir-28	AAGGAGCTCACAGTCTATTGAG(AAGGAGCTCACAGTCTATTGAG)	0,059	*0,007*	0,058	*0,008*
hsa-mir-26b	TTCAAGTAATTCAGGATAGGT(TTCAAGTAATTCAGGATAGGTT)	0,057	*0,000*	0,074	*0,019*
hsa-mir-500a	ATGCACCTGGGCAAGGATTCTG(TAATCCTTGCTACCTGGGTGAGA)	0,054	*0,014*	0,078	*0,012*

Table S2 (Related to Table 1). Binding affinities (K_D, µM) of compounds PA-1, PA-2 and PA-3 for pre-miR-372 alone or in the presence of 100 eq. of tRNA (K'_D, µM) and DNA (K''_D, µM).

Name	K_D[a]	K'_D tRNA[b]	K'_D/K_D	K''_D DNA[c]	K''_D/K_D
**PA-1**	0.15	0.14	1.0	0.17	1.1
**PA-2**	0.53	0.62	1.2	5.8	11
**PA-3**	1.15	1.13	1.0	1.15	1.0

[a] Binding studies were performed on 5'-FAM-pre-miR-372 in buffer A (20 mm Tris–HCl (pH 7.4), 12 mm NaCl, 2.5 mm $MgCl_2$, and 1 mm DTT). [b] Measured in the presence of a 100-fold excess of a mixture of natural tRNAs (tRNA mix). [c] Measured in the presence of a 100-fold excess of a 15-mer duplex DNA. All K_D values are given with an uncertainty of ±10%.

Table S3 (Related to Table 1). IC_{50} values (µM)[a] for the inhibition of pre-miR-372 maturation in the presence of recombinant Dicer enzyme or HEK93 cell lysates.

Name	IC_{50} (µM) (recombinant Dicer)	IC'_{50} (µM) (cell lysates)	IC'_{50}/IC_{50}
**1**	1.06	2.88	2.7
**2**	16.3	65.4	4.0
**3**	12.7	34.4	2.7

[a] IC_{50} experiments were performed in the presence of 50 nM of 3'-dabcyl-5'-FAM-pre-miR-372 beacon and 0.25U of human recombinant Dicer in Buffer A and represent the average of three independent experiments. All IC_{50} values are given with an uncertainty of ±10%.
